# *De novo* transcriptome analyses provide insights into opsin-based photoreception in the lanternshark *Etmopterus spinax*

**DOI:** 10.1371/journal.pone.0209767

**Published:** 2018-12-31

**Authors:** Jérôme Delroisse, Laurent Duchatelet, Patrick Flammang, Jérôme Mallefet

**Affiliations:** 1 University of Mons (UMONS), Research Institute for Biosciences, Biology of Marine Organisms and Biomimetics, Mons, Belgium; 2 Catholic University of Louvain (UCLouvain), Earth and Life Institute, Marine Biology Laboratory, Louvain-La-Neuve, Belgium; University of Ferrara, ITALY

## Abstract

The velvet belly lanternshark (*Etmopterus spinax*) is a small deep-sea shark commonly found in the Eastern Atlantic and the Mediterranean Sea. This bioluminescent species is able to emit a blue-green ventral glow used in counter-illumination camouflage, mainly. In this study, paired-end Illumina HiSeq^TM^ technology has been employed to generate transcriptome data from eye and ventral skin tissues of the lanternshark. About 64 and 49 million Illumina reads were generated from skin and eye tissues respectively. The assembly allowed us to predict 119,749 total unigenes including 94,569 for the skin transcriptome and 94,365 for the eye transcriptome while 74,753 were commonly found in both transcriptomes. A taxonomy filtering was applied to extract a reference transcriptome containing 104,390 unigenes among which 38,836 showed significant similarities to known sequences in NCBI non-redundant protein sequences database. Around 58% of the annotated unigenes match with predicted genes from the Elephant shark (*Callorhinchus milii)* genome. The transcriptome completeness has been evaluated by successfully capturing around 98% of orthologous genes of the « Core eukaryotic gene dataset » within the *E*. *spinax* reference transcriptome. We identified potential “light-interacting toolkit” genes including multiple genes related to ocular and extraocular light perception processes such as opsins, phototransduction actors or crystallins. Comparative gene expression analysis reveals eye-specific expression of opsins, ciliary phototransduction actors, crystallins and vertebrate retinoid pathway actors. In particular, mRNAs from a single rhodopsin gene and its potentially associated peropsin were detected in the eye transcriptome, only, confirming a monochromatic vision of the lanternshark. Encephalopsin mRNAs were mainly detected in the ventral skin transcriptome. In parallel, immunolocalization of the encephalopsin within the ventral skin of the shark suggests a functional relation with the photophores, *i*.*e*. epidermal light-producing organs. We hypothesize that extraocular photoreception might be involved in the bioluminescence control possibly acting on the shutter opening and/or the photocyte activity itself. The newly generated reference transcriptome provides a valuable resource for further understanding of the shark biology.

## Introduction

Over the past 450 million years, cartilaginous fish have evolved to fill a large range of predatory niches in marine and freshwater ecosystems [1, 2]. The development of a sophisticated battery of sensory systems is considered as an important factor explaining the evolutionary success of the elasmobranchs and their relatives [2, 3]. Sharks have been considered as “swimming noses” because of their high olfactory abilities. Their large telencephalon, *i*.*e*. the forebrain, is indeed primarily dedicated to olfaction [4, 5]. Other sensory systems—including light perception–have received traditionally much less attention [6, 7]. Early studies reported that the retina of the majority of cartilaginous fishes contains only rod photoreceptors [8, 9]. These organisms were thought to have poor visual acuity with eyes that are specialized for scotopic vision (*i*.*e*., dim light condition) with no capacity for photopic vision (*i*.*e*., bright light condition) or color discrimination [4]. Rods indeed serve scotopic vision and are highly sensitive, at the expense of visual acuity. Other specializations include (*i*) the presence of a tapetum at the rear of the eye for reflecting light back on to the photoreceptors and (*ii*) a high photoreceptor to ganglion cell summation ratio that increases sensitivity at the expense of acuity [8]. More recently, it was demonstrated that the majority of cartilaginous fishes are able to function under a range of photopic and scotopic light intensities and actually possess a duplex retina containing both rod and cone photoreceptors [7, 10–14]. Cones are used for photopic and color vision and are responsible for higher visual acuity. Some deep-sea sharks and rajids appear to have all-rod retinas [15–17].

Photoreceptors contain visual pigments made up of membrane proteins, the so-called opsins, linked to a chromophore prosthetic group, which changes its conformation when exposed to light, inducing a cascade that finally transmits the visual information to the brain [7]. The opsin chromophore is a vitamin A-based retinaldehyde, either the retinal (A1) or the 3,4-dehydroretinal (A2) in fish [7]. Most shark species, mainly epipelagic, possess A1-associated opsins sensitive to blue green light (historically called “rhodopsins” while this term is now used as a generic term to describe all visual pigments). Most deep-water sharks also have A1-associated opsins sensitive to deep blue light (historically called chrysopsins) while some freshwater species have A2-associated opsins that have a red shift in their absorbance maxima (historically called porphyropsins) [4, 7, 16].

In parallel to the visual system, photoreceptor cells can also be involved in non-image-forming light detection. The research on extraocular photoreception was pioneered by Steven and Millott [18–20]. The diffuse photosensitivity over the whole or parts of the animal’s skin was described as the “dermal light sense” but even deeper tissues of the body, such as neural or brain cells, can be photosensitive [18–22]. The photoreceptors present outside the eyes are referred to as extraocular or extraretinal [23, 24]. Like the visual photopigments, non-visual photopigments may consist of an opsin protein linked to a retinal chromophore. Extraocular photoreception can play important roles in the behavior and the physiology of animals [18–20, 24]. In sharks, extraocular photoreceptors are commonly known to be associated to the pineal gland [25].

Shark opsin diversity has been extensively investigated using the sequenced genome of the elephant shark, *Callorhinchus milii* [26–28]. Unusually for a deep-sea fish, this species possesses cone pigments and the potential for trichromacy. The genome encodes for four visual opsins: a visual rhodopsin (RHO1) and three color visual opsins (*i*.*e*., middle wavelength-sensitive, RHO2; long wavelength-sensitive, LWS1 and LWS2) [26, 28, 29]. More surprisingly, the genome also encodes for 13 non-visual opsins: a pinopsin, a parapinopsin, a RGR-opsin, two TMT-opsins (*i*.*e*., teleost multiple tissue opsin), a VA-opsin (*i*.*e*., vertebrate-ancient opsin), an encephalopsin (also designated as panopsin), a peropsin, three neuropsins and two melanopsins (*i*.*e*, non-visual rhabdomeric opsin) [26–28]. This study provides the most complete opsin dataset in a cartilaginous fish to date.

**Fig 1:**
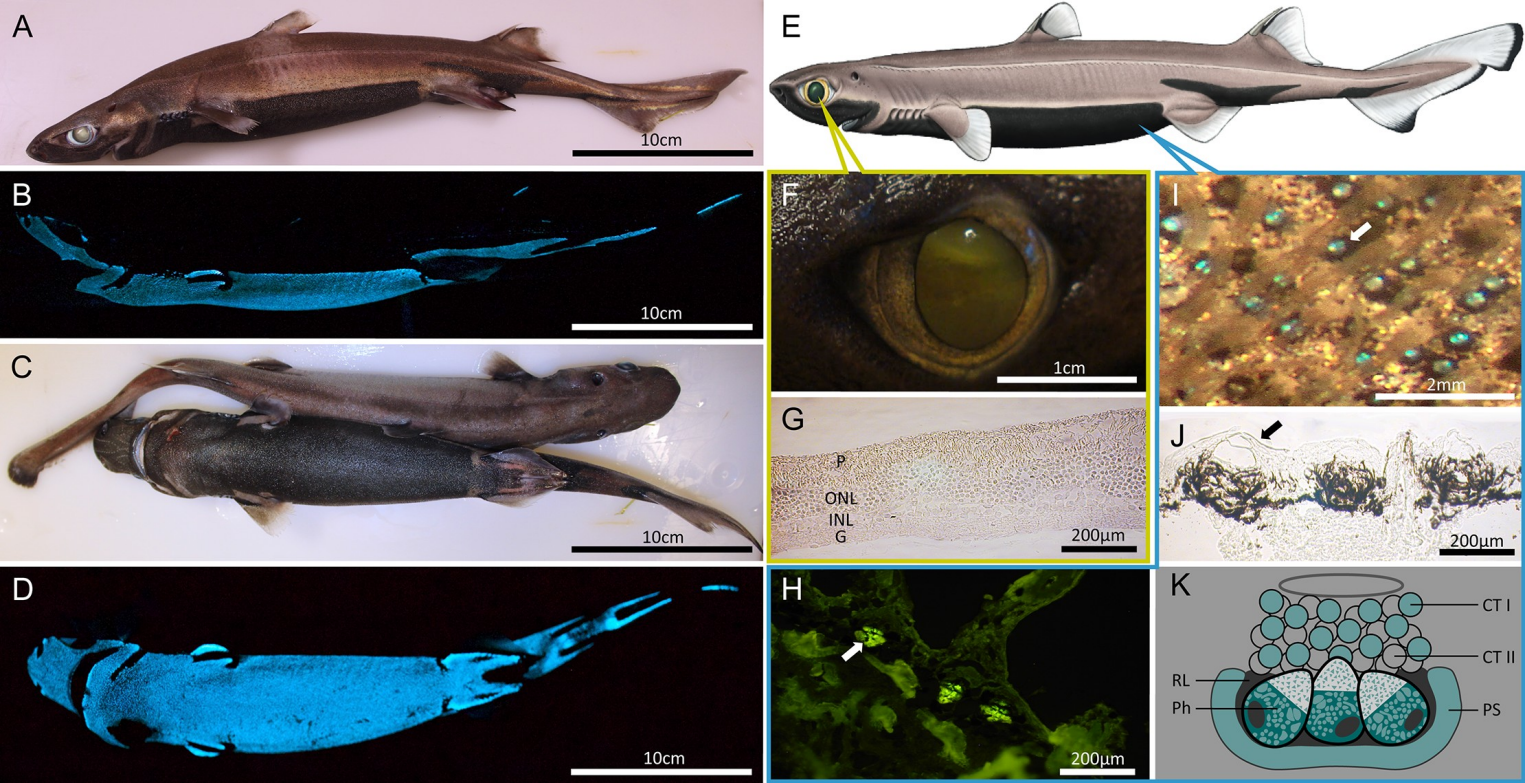
The lanternshark *Etmopterus spinax*. A-B, E: lateral views of the shark (2018 Shark Trust, www.sharktrust.org). B: lateral bioluminescence emission pattern. C: ventral and dorsal views of the shark. D: ventral bioluminescence emission pattern. F: Eye of the shark. G: histological section through the shark retina. H, J: histological sections through the shark skin. I: in vivo observation of ventral skin photophores, K: Schematic reconstruction of a photophore (modified from [95, 96]). Annotations: C: connective tissue, CTI: cellular type I, CTII: cellular type II, D: denticle, L: lens, G: ganglionic layer, E: epidermis, INL: inner nuclear layer, Ir: iris, ONL: outer nuclear layer, P: pigmented layer, Ph: photocyte, PS: pigmented shield, RL: reticulated layer.

The velvet belly lanternshark *Etmopterus spinax* (Linnaeus, 1758) is a common deep-sea shark occurring along the continental shelf of the Eastern Atlantic Ocean and in the Mediterranean Sea [3, 30]. This species is able to emit a blue-green ventral glow (λmax = 486 nm) thanks to thousands of tiny photophores spread in the ventral epidermis [31–33]. Photophores are composed of a cluster of photogenic cells, the photocytes, enclosed in a pigmented sheath and surmounted by a lens. Some pigmented cells playing an iris-like role are also located between the lens and the photocytes [31, 32] (**Fig 1**). *E*. *spinax* has been used recently as a model species for experimental studies on the physiological control of its natural luminescence [34–37]. However, it has been poorly investigated from the molecular point of view and functional molecular data on this species are absent from public databases. Here, we report the first transcriptome data for the velvet lanternshark *E*. *spinax*. *De novo* RNA sequencing was performed on the tapeta-equipped eye containing the all-rod retina [33] and on ventral integument tissues of the shark, *i*.*e*. main light emitting area of the shark. The aim of this study was to investigate the opsin-based ocular and extraocular photoreception of the lanternshark *E*. *spinax*. We highlighted multiple actors of the opsin-based phototransduction cascade in ocular and extraocular tissues as well as other “light-interacting actors” [38]. Our results support the idea that the lanternshark receives and integrates constant light information from the environment but also possibly from their own luminous organs. Light reception at the level of a bioluminescent organ could be linked to a specific control of the light emission at the level of the photophore as suggested in various other luminous metazoans (*i*.*e*., in *Amphiura* [Echinodermata], *Mneniopsis* [Ctenophora], or *Sepiola* [Mollusca]) [39–44].

## Material and methods

### Tissue Collection and preparation, ethics statement & RNA isolation

Adult velvet belly lanternsharks, *E*. *spinax* were captured by long-lines lowered at 200 m depth in the Raunefjord, Norway (60°169 N; 05°089 E) (see also [31, 32] for more details) during multiple field sessions between August 2014 and January 2016. Living sharks were kept at Bergen University Marine Station (Espegrend, Norway) in a seawater tank (1m^3^) filled with cold (6° C) running seawater pumped from the depths of the adjacent fjord. The tank was placed in a dark room to keep animals under good physiological conditions. The shark collection and experiments were performed following the local instructions for experimental fish care (PERMIT” number 12/14048). Following the local instructions for experimental fish care, 6 captive animals were euthanized by a blow to the head followed by a full incision of the spinal cord at the back of the head. Animal procedures were conducted in compliance with the Belgian national guidelines and in agreement with the European directive 2010/63/UE, under the approval of the Animal Ethics Committee of the Catholic University of Louvain in Louvain-la-Neuve. One individual was used for the transcriptomic approach.

The global methodological pipeline of the study is illustrated in the **Fig 1**. Shark tissues from one shark individual were dissected and directly frozen in liquid nitrogen. Pieces of eye and skin tissues were then permeabilized in RNA*later*^TM^*-*Ice (Life Technologies) during one night at -20°C following the manufacturer’s instructions and then stored at -80°C or directly processed for RNA extraction. Total RNA was extracted following the Trizol reagent-based method. The quality of the RNA extracts was checked by gel electrophoresis on a 1.2 M TAE agarose gel, and by spectrophotometry using a Nanodrop spectrophotometer (LabTech International). The quality of the RNA was also assessed by size-exclusion chromatography with an Agilent Technologies 2100 Bioanalyzer.

In parallel, patches of ventral and dorsal skin as well as eye of the shark were removed and either fixed in 4% paraformaldehyde phosphate buffer saline (PBS) for 12 hours at 4°C and stored at 4°C in PBS until use or directly frozen at -80°C without any treatment. Fixed pieces of ventral and dorsal skin (1 cm^2^) were used to perform histological and immunohistochemical analyses while frozen samples were used to perform immunoblots.

### cDNA Library preparation and sequencing

cDNA library preparation and sequencing were performed by the Beijing Genomics Institute (BGI, Hong Kong) according to the manufacturer’s instructions (Illumina, San Diego, CA, USA) and following the same procedure described in [45, 46]. High-throughput sequencing was conducted using the Illumina HiSeq^TM^ 2000 platform to generate 100-bp paired-end reads.

### *De novo* assembly and read mapping

A reference *de novo* transcriptome assembly was performed from *E*. *spinax* reads derived from eye and skin tissues. Before the transcriptome assembly, the raw sequences were filtered to remove the low-quality reads. The filtering steps were as follows: 1) removal of reads containing only the adaptor sequence; 2) removal of reads containing over 5% of unknown nucleotides ‘‘N”; and 3) removal of low quality reads (those comprising more than 20% of bases with a quality value lower than 10). The remaining clean reads were used for further analysis. Quality control of reads was accessed by running the FastQC program [47].

Transcriptome *de novo* assembly was carried out with short paired-end reads using the Trinity software [48] (*version release-20121005; min_contig_length 100*, *group_pairs_distance 250*, *path_reinforcement_distance 95*, *min_kmer_cov 2*). After Trinity assembly, the TGI Clustering Tool (TGICL) [49] followed by Phrap assembler (http://www.phrap.org) were used for obtaining distinct sequences. These sequences are defined as unigenes. Unigenes, that are here defined as non-redundant assembled sequences obtained from assembly and/or clustering [50], can either form clusters in which the similarity among overlapping sequences is superior to 94%, or singletons that are unique unigenes.

As the length of sequences assembled is a recognized criterion for assembly success in terms of contiguity, we calculated the size distribution of both contigs and unigenes. To evaluate the depth of coverage, all usable reads were realigned to the unigenes using SOAP aligner with the default settings [51].

For both transcriptomes, unigene expression was evaluated using the “Fragments per kilobase of transcript, per million fragments sequenced” (FPKM) method. The FPKM value is calculated following the specific formula $FPKM=\frac{10^{6}C}{N.L/10^{3}}$ where C is the number of fragments showed as uniquely aligned to the concerned unigene, N is the total number of fragments that uniquely align any unigene, and L is the base number in the coding DNA sequence of the concerned unigene. The FPKM method integrates the influence of different gene length and sequencing level on the calculation of gene expression.

### Functional gene annotation of *E*. *spinax* transcriptome

**Fig 2:**
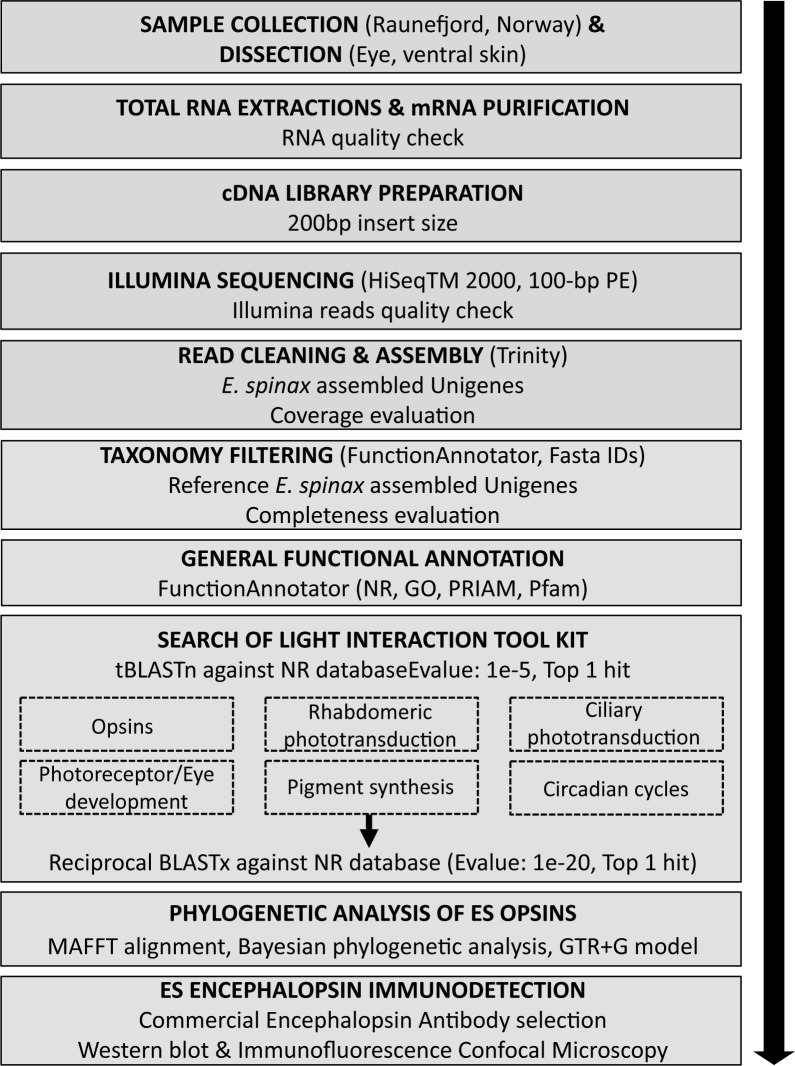
Methodological pipeline of the study performed on the lanternshark *E*. *spinax*.

Following the pipeline described in the **Fig 2**, all unigenes were used for homology searches against the NCBI non-redundant protein sequences (NR) database using the LAST algorithm implemented in FunctionAnnotator. Based on NR annotation, taxonomic distribution analyses were performed with FunctionAnnotator [52]. In order to generate a high-confidence *E*. *spinax* reference transcriptome, and eliminate sequences from bacteria and/or non-metazoans (*i*.*e*., potential contaminations, symbiotic organisms…), taxonomy filtering has been performed based on taxonomic distribution results (*i*.*e*., sequences deriving from Eukaryotes, excluding Plantae, were selected).

To annotate the reference transcriptome, all unigenes were used for homology searches against various databases such as NCBI NR (LAST algorithm), PRIAM (RPS-BLAST algorithm) and PFAM (RPS-BLAST algorithm) using FunctionAnnotator (E-value < 1e-5) [52]. The Blast2GO pipeline (b2g4pipe) [53] was also used to get Gene Ontology annotation according to molecular function, biological process and cellular component ontologies (http://www.geneontology.org) from NR annotation results.

The completeness of the transcriptomes was evaluated using tBLASTn search for the 456 human transcripts, from the « Core Eukaryotic Gene » dataset, that are highly conserved in a wide range of eukaryotic taxa and has been previously used to assess the quality of genomes and transcriptomes (http://korflab.ucdavis.edu/datasets/cegma/) [54].

### Detection of opsins and “light interacting toolkit” genes in *E*. *spinax*

In order to identify genes involved in light-mediated processes such as phototransduction (*i*.*e*., opsins, actors involved in the phototransduction cascade associated to rhabdomeric or ciliary opsins), photoreceptor specification, eye development/retinal determination network, retinoid pathway, melanin pigment synthesis, crystallins, diurnal clock and circadian cycles, potential transcripts of interest were selected based on the phylogenetically-informed annotation (PIA) tool developed to search for light-interacting genes in transcriptomes of non-model organisms [38]. For specific opsin searches, the PIA dataset was implemented with various reference metazoan opsin sequences based on [55] to cover the whole opsin diversity. First, the “Light Interaction Genes” were searched in the newly generated reference transcriptome of *E*. *spinax* using *BLAST* analyses (1 hit, E-value < 1e-20). All individual unigenes retrieved were then reciprocally searched in the NR databases (GenBank, RefSeq, EMBL, DDBJ, PDB databases) using tBLASTn (with 1 hit maximum) implemented in Geneious (v.8.1.9) [56].

Phototransduction, in particular, is a biochemical process by which the photoreceptor cells generate electrical signals in response to captured photons. Two main phototransduction cascades characterize visual rhabdomeric and ciliary photoreceptors of metazoans [57, 58].

Ciliary photoreceptors, classically associated with vertebrate eyes, employ a phototransduction cascade that includes ciliary opsins. The vertebrate cascade starts with the absorption of photons by the photoreceptive C-opsins (*e*.*g*., rho). Opsin activation triggers hydrolysis of cGMP by activating a transducing phosphodiesterase 6 (*e*.*g*., Pde6) cascade via the GTP-binding protein Gi/Gt/(Go) protein alpha subunit (*e*.*g*., Gnat1) (Go protein-mediated phototransduction cascades were also reported in ciliary visual cells of scallop [59] as well as in amphioxus [60] and lizard parietal eye [61]), which results in closure of the cGMP-gated cation channels (*i*.*e*., Cnga1) in the plasma membrane and membrane hyperpolarization. The hyperpolarization of the membrane potential of the photoreceptor cell modulates the release of neurotransmitters towards downstream cells. Recovery from light involves the deactivation of the light-activated intermediates: photolyzed opsin is phosphorylated by rhodopsin kinase (*i*.*e*., Grk1) and subsequently capped off by arrestin (*e*.*g*., Sag); GTP-binding transducin alpha subunit (*e*.*g*., Gnat1) deactivates through a process that is stimulated by the regulator of G protein signaling 9 (*i*.*e*., Rgs9). Recoverin (*i*.*e*., Rcvrn) inhibits phosphorylation of rhodopsin [62] by binding to rhodopsin kinase [63, 64].

Rhabdomeric photoreceptors, classically associated with invertebrate eyes, employ a cascade involving R-opsins, G protein alpha q (*i*.*e*., Gnaq), phospholipase C (*i*.*e*., Plcb4) and transient receptor potential ion channels (*i*.*e*., TRP, TRPL). Visual signaling is initiated with the activation of R-opsin by light. Upon absorption of a light photon the opsin chromophore is isomerized which induces a structural change that activates the opsin. The photoconversion activates heterotrimeric Gq protein via GTP-GDP exchange, releasing the G alpha q subunit. G alpha q activates the phospholipase C (*i*.*e*., Plcb4), generating IP3 and DAG from PIP2. DAG may further release polyunsaturated fatty acids (PUFAs) via action of DAG lipase. This reaction leads to the opening of cation-selective channels (*i*.*e*., TRP) and causes the depolarization of the photoreceptor cells.

Ciliary and Rhabdomeric cascades can be deactivated by arrestins (*i*.*e*., Sag, Arr1) and rhodopsin kinases (*i*.*e*., Grk1, Grk4/5/6) and regenerated by retinal binding proteins [43, 44].

Reference genes associated with all light-mediated processes are listed in the **S1 Table**. Blast hits with significant E-values strongly indicate homologous proteins. In parallel, searches were performed on two chondrichthyan reference genomes: *Rhincodon typus* (22 March 2017; predicted proteins; 27,896 sequences; 13,150,867 total letters) and *Callorhinchus milii* (12 May 2014; predicted proteins; 28,237 sequences; 17,563,624 total letters).

### Opsin characterisation and phylogenetic analyses

For all putative opsin candidates, secondary structure prediction–in particular, of the transmembrane helices–was performed using the MENSAT online tool [65–67]. *In silico* translation (ExPASy translate tool, http://expasy.org/tools/dna.html) was performed on the opsin-like sequences retrieved from the *E*. *spinax* transcriptomes. A multiple alignment of the amino-acid sequences of the putative opsins was performed using MAFFT algorithm using the consistency-based iterative refinement method E-INS-i [68] (implemented in Geneious [56]). Aligned residues were highlighted by similarity group conservation (*i*.*e*., RasMol color option) and similarity comparisons were calculated in SIAS website platform (http://imed.med.ucm.es/Tools/sias.html). Sequence alignments made it possible to identify opsin characteristic features such as the lysine residue involved in the Schiff base linkage, the counterion, the amino acid triad present in the helix involved in the G protein contact, or putative disulfide bond sites. The predicted molecular weight of the opsins was calculated using the “Compute pI/Mw tool” on the ExPASy Proteomics Server [69, 70].

For phylogenetic analyses, reference opsin sequences from metazoan species were added in the MAFFT alignment. Sequences of non-opsin GPCR receptors (*i*.*e*., melatonin receptors) were also added and chosen as outgroup following previous reference studies [33, 71–73]. In total, 96 sequences were used for the phylogenetic analysis (**S2 Table**). The alignment was trimmed with the BMGE software (http://mobyle.pasteur.fr/cgi-bin/portal.py) [74] using default parameters in order to keep the conserved 7TM core of the proteins and discard N-terminal and C-terminal sequence extremities to avoid unreliably aligned regions (final alignment of 322 characters). We performed a Bayesian phylogenetic analysis with MrBayes v.3.2 software [75] using the GTR+G model based on recent opsin studies [33, 71–73]. Four independent runs were performed, until a standard deviation value inferior to 0.01 was reached (after 3,500,000 generations).

### Encephalopsin immunodetection

We used a commercial polyclonal antibody directed against human encephalopsin (anti- *H*. *sapiens* encephalopsin Pab, Genetex, GTX 70609, lot number 821400929) to immunolocalize the encephalopsin of *E*. *spinax*. For immunohistochemistry, fixed eyes and skin patches were bathed in PBS with increasing sucrose concentration: 10% for 1 h, 20% for 2 h, and finally 30% sucrose overnight. Tissues were then embedded in O.C.T. compound (Tissue-Tek, The Netherlands) and quickly frozen in isopentane chilled with liquid nitrogen. Thin sections were cut with a cryostat microtome (CM3050 S, Leica, Germany) and collected on coated slides (Superfrost, Thermo scientific). Sections were blocked with TTBS (Trizma base (Sigma) 20 mM, NaCl 150 mM, pH 7,5 + 1% Tween 20 (Sigma)) containing 5% BSA (Amresco). They were then incubated overnight at 4°C with the anti-encephalopsin antibody diluted 1:400 in TTBS 5% BSA. Visualization of encephalopsin immunoreactivity was done after a 1 h incubation of the sections at RT with fluorescent dye labeled secondary antibody (Goat Anti-Rabbit, Alexa Fluor 594, Life Technologies Limited) diluted 1:200 in TTBS 5% BSA. In order to label the nucleus of each cell, sections have been subject to a DAPI (DAPI nucleic acid stain, Invitrogen) staining during 15 min before being mounted (Mowiol 4–88, Sigma). Sections were examined using an epifluorescence microscope (Polyvar SC microscope, Leica Reichter Jung) equipped with a Nikon DS-U1 digital camera coupled with NIS-elements FW software. Control sections were incubated in TTBS 5% BSA with no primary antibody.

For Western blot analyses, proteins were extracted from frozen tissue samples using a two-step protocol at 4°C. Samples (size: 1 cm x 3 cm) were homogenized in 1000 μl of TEN buffer (10 mM Tris, pH 7,5; 1 mM EDTA, pH 8,0; 100 mM NaCl) supplemented with protease inhibitors (complete–Mini tablets, Roche). The extract was sonicated and centrifuged at 800g for 10 min. The supernatant was discarded and the pellet was re-extracted with 200 μl of TEN buffer containing 10% NP-40 and 0,25% SDS (10 mM Tris, pH 7,5; 1 mM EDTA, pH 8,0; 100 mM NaCl; 0,5% NP-40; 0,25% SDS; 0,5% Deoxycholate) with protease inhibitors. After sonication and centrifugation (15 min, 100 000 g), the supernatant was collected. Protein concentration in each extract was measured using Pierce^TM^ BCA Protein Assay Kit (Thermo Scientific). Laemmli buffer (Biorad) and β-mercaptoethanol (βMSH, Biorad) were added to each protein extract and the proteins were electrophoretically separated at 200 V for 35 min on 12% SDS-PAGE gels. The separated proteins were then electroblotted on a nitrocellulose membrane. Membrane was incubated overnight with the primary anti-encephalopsin antibody and with secondary antibody (ECL HRP conjugated anti-rabbit antibody, Life Sciences, NA934VS, lot number 4837492) for 1 h. Antibody detection was performed with the reagents of the detection kit (HRP Perkin-Elmer, NEL 104) following the manufacturer instructions. The dilution for the primary antibody was 1:2000. In order to determine the specificity of the observed band, control experiments were included: (*i*) omission of the primary antibody and (*ii*) validation of membrane protein extraction and western blot protocols using an anti-cadherin (*i*.*e*., a very abundant protein involved in cell adhesion [76, 77]) antibody (Purified Mouse Anti-E-Cadherin (BD Transduction Laboratories, 610181).

## Results

### Illumina transcriptome sequencing and *de novo* assembly

In total, 49,178,512 and 64,000,000 raw reads, with a length of 100bp, were generated from a 200bp insert library from the eye and ventral skin libraries, respectively. Dataset qualities were checked using the FastQC software. The datasets of raw reads were deposited in NCBI database under SRA experiment number SRP153043 (SRX4379544, SRX4379543). After low quality reads filtering, the remaining high quality reads (*i*.*e*., 46,012,442 for eye transcriptome and 51,160,110 for ventral skin transcriptome) were used to assemble the eye and ventral skin transcriptomes with the Trinity software. According to the overlapping information of high-quality reads, contigs were generated. For eye transcriptome data, the average contig length was 291 bp and the N50 (*i*.*e*., the median contig size) was of 545 bp. For ventral skin transcriptomic data, the average contig length was 227 bp and the N50 was of 316 bp. Q20 percentages (base quality more than 20) were superior to 95% for both datasets. The GC percentage is around 47% for both transcriptomes.

Using paired-end joining and gap filling, contigs were further assembled into 94,365 unigenes, *i*.*e*. non-redundant unique sequences, for the eye dataset and 93,569 for the ventral skin dataset with a total of 119,749 different unigenes. Eye transcriptome unigenes include 23,183 clusters and 71,182 singletons. Ventral skin transcriptome unigenes contain 14,811 clusters and 78,758 singletons. The size distributions of contigs and unigenes are shown in **S1 Fig** and numerical data are summarized in **Tables 1** and **2**.

**Table 1:** Description of the output sequenced data. Q20 percentage is the proportion of nucleotides with quality value larger than 20 in reads. GC percentage is the proportion of guanidine and cytosine nucleotides among total nucleotides.

***E*. *spinax* tissue samples**	**Eye**	**Ventral skin**
**Total Raw Reads**	49,178,512	64,000,000
**Total Clean Reads**	46,012,442	51,160,110
**Total Clean Nucleotides (nt)**	4,601,244,200	5,116,011,000
**Q20 (%)**	97.99	95.99
**GC (%)**	47.15	46.31

**Table 2:** Summary statistics of assemblies for *E*. *spinax* eye and ventral skin transcriptomes.

**Assemblies**		**Number**	**Total Length (nt)**	**Mean Length (nt)**	**N50 (nt)**	**Distinct Clusters**	**Distinct Singletons**
**Eye**	Contig	307,547	89,448,805	291	545	-	-
	Unigene	94,365	91,409,720	969	1975	23,183	71,182
**Ventral skin**	Contig	321,838	73,177,644	227	316	-	-
	Unigene	93,569	50,577,046	541	665	14,811	78,758
**Pooled**	Unigenes	119,749	93,903,071	784	1412	27,526	92,223
**Taxonomy filtered**	Unigenes	104,390	87,719,452	840	1558	26,955	77,435

**Fig 3:**
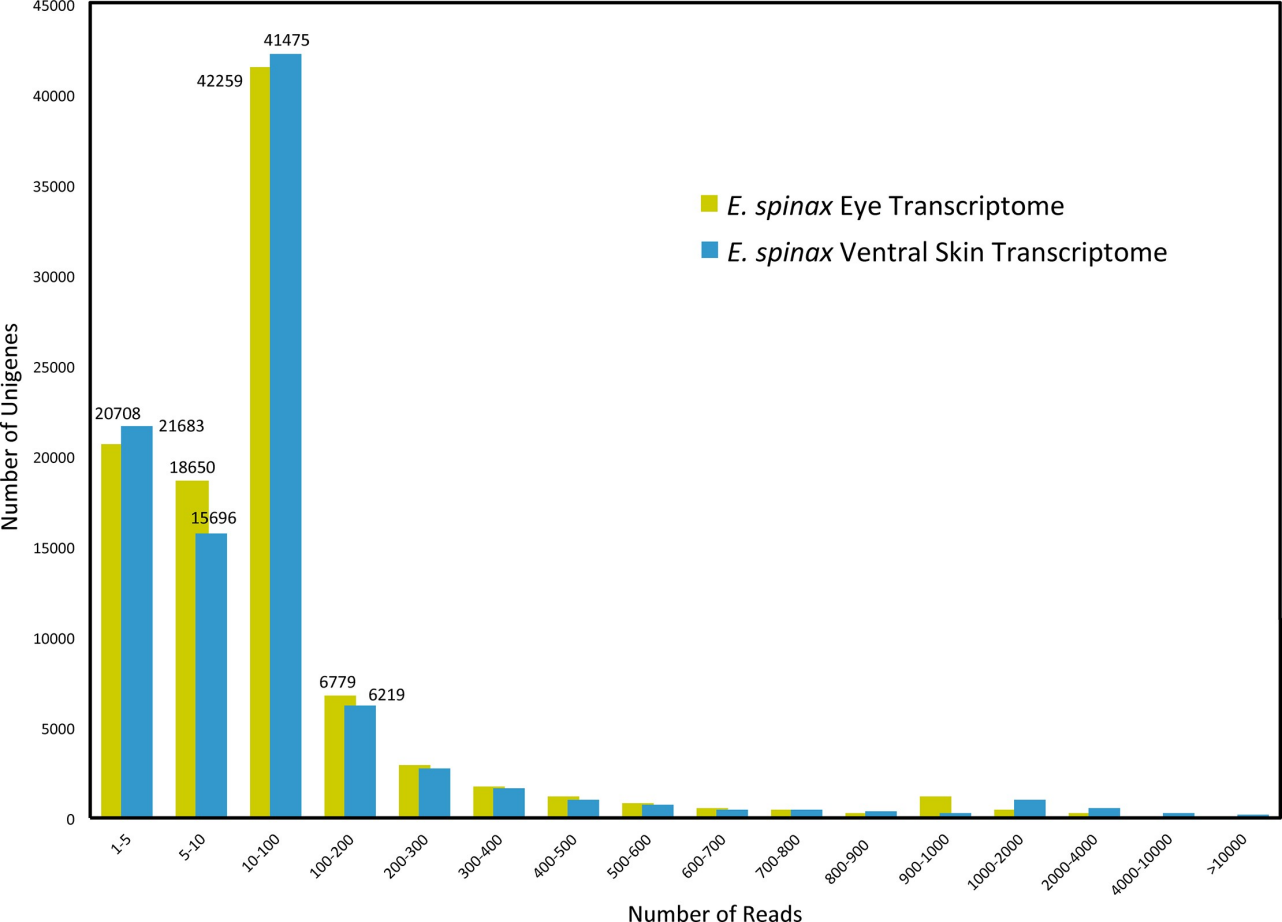
Distribution of the assembled *E*. *spinax* unigenes in function of the number of reads to which they can be aligned. The x-axis represents the « number of reads » classes.

To evaluate the coverage of the two transcriptomes, all the usable sequencing reads were realigned to the all unigenes. More than 78% of eye transcriptome unigenes and more than 76% of ventral skin transcriptome unigenes were realigned with more than 5 reads (**Fig 3**) indicating a good coverage.

**Fig 4:**
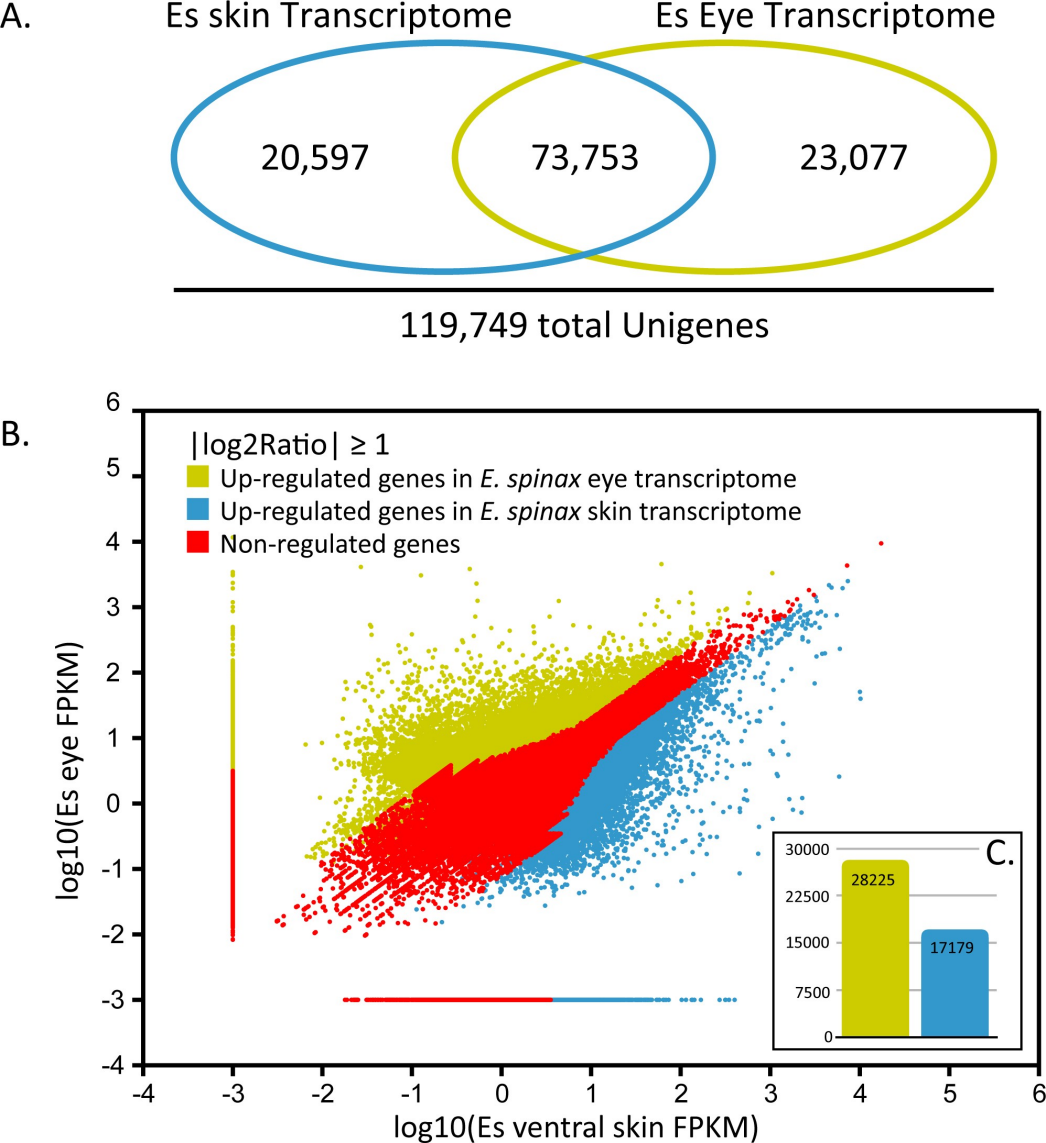
Comparative gene expression in *E*. *spinax* eye and ventral skin tissues.

On a total of 119,749 predicted unigenes, 20,597 were found in skin transcriptome and 23,077 in eye transcriptome while 73,753 were detected in both transcriptomes (**Fig 4A**). For descriptive purpose, a comparative gene expression analysis was performed by mapping FPKM values (*i*.*e*., log_10_(FPKM value ventral skin transcriptome) against log_10_(FPKM value eye transcriptome), calculated for all predicted unigenes (**Fig 4B)**. However it has to be clarified that the transcriptome data have been generated in the purpose of new gene discovery, not differential expression analyses, as no biological or technical replication was performed as a part of the study. Based on the “|log2Ratio|≥1” threshold, 28,225 unigenes were found to be upregulated in the eye transcriptome and 17179 in the ventral skin transcriptome (**Fig 4C**).

### Function annotation of *E*. *spinax* transcriptome

On all the 119,749 *E*. *spinax* pooled unigenes, 54,196 (45,3%) show significant matches to the NCBI NR database. Because of the lack of genome reference in *E*. *spinax* and, possibly, the relatively short length of some unigene sequences, 44,7% of the assembled sequences could not be matched to any known genes. Among annotated unigenes from the pooled transcriptome, 22,387 sequences were matched to the elephant shark *Callorhinchus milii* (41%, **S2A Fig**) for which the genome has been sequenced.

**Fig 5:**
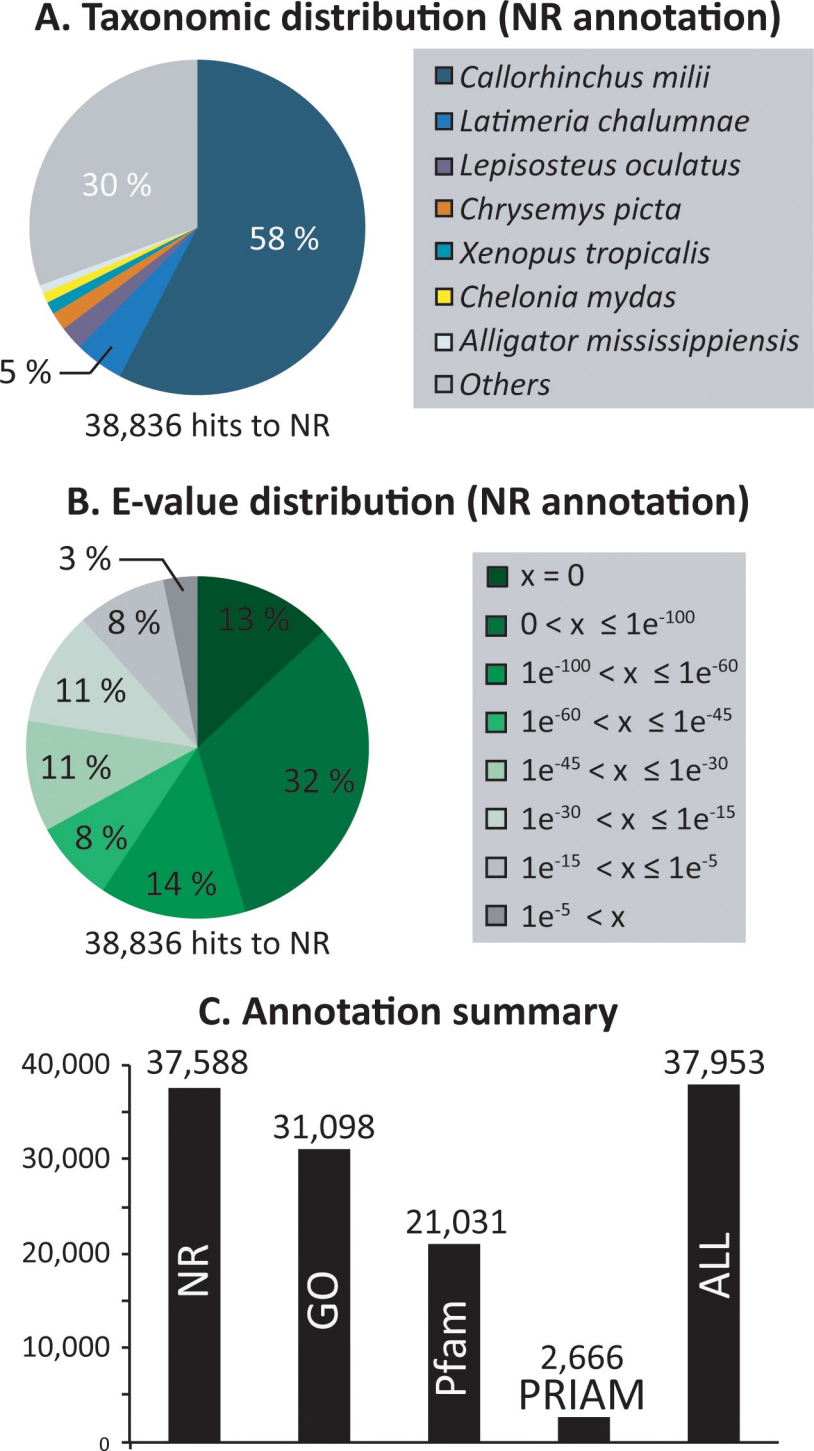
Species distribution, E-value distribution and annotation summary of the taxonomy-filtered reference transcriptome of *E*. *spinax*. A. Species distributions of the top BLAST hits for all unigenes from *E*. *spinax* taxonomy-filtered reference transcriptome in the NR database. B: E-value distributions of NR annotation results. C. Distribution of annotation results. Unigenes of *E*. *spinax*, from the taxonomy-filtered reference transcriptome, were annotated using the NR, GO, PRIAM and Pfam databases (see text for details).

Taxonomy distribution analyses revealed the presence of contaminations within the ventral skin transcriptome (*e*.*g*., *Bradyrhizobium* sp, *Hordeum* sp) (**S2B Fig**). Based on these results, an additional filtration step was performed to eliminate contaminants such as bacterial and plant sequences (*i*.*e*., the unigenes that match to non-Eukaryotes or Plantae were eliminated. Remaining sequences (*i*) have matches to non-Plantae Eukaryotes or (*ii*) do not have any match). The summary statistics of the taxonomy filtered reference transcriptome assembly are presented in **Table 2**. The main represented species within the unigene annotation of the reference transcriptome is the elephant shark *Callorhinchus milii* (58%), followed by *Latimeria chalumnae* (5%) (**Fig 5A**). The genome of the whale shark *Rhincodon typus* was recently published [78] but is not yet implemented in the NR database version used by the webtool FunctionAnnotator [52]. On the 104,390 *E*. *spinax* unigenes present in the filtered reference transcriptome, 37,952 show significant matches to molecular databases: 37,588 to NR (37.2%, E-value > 1e^-5^), 31,098 to GO, 2,666 to PRIAM (E-value > 1e^-5^), 21,031 to Pfam (E-value > 1e^-5^) (**Fig 5C**).

The completeness of the transcriptome was evaluated by searching genes from the « Core eukaryotic gene dataset » within the taxonomy filtered reference transcriptome of *E*. *spinax* [54]. A total of 451 (98.9%) of the 456 highly conserved CEGs were detected (E-value < 1e-5). Annotation results are summarized in the **Fig 5**.

The annotation success was estimated by ranking the annotation E-values results obtained from the NR database comparison. E-value distributions are presented in **Fig 5**. More than 78% of annotation results have an E-value inferior to 1e^-30^.

On the basis of the NR annotation, the Blast2go software was used to obtain Gene Ontology annotation of the assembled unigenes, and then the GO functional classifications of the unigenes were performed. For all *E*. *spinax* unigenes, in total, 31,098 unigenes with BLAST matches to known proteins were assigned to GO classes. Specific GO categories related to the light perception process, including “Visual perception” (19 hits, GO:0042574), “Phototransduction” (8 hits, GO:0016918), “Retinal binding” (32 hits, GO:0007602) and “Retinal metabolic process” (318 hits, GO:0007601) were targeted in the *E*. *spinax* pooled transcriptome (data not shown) indicating the expression of phototransduction actors.

The FPKM method was used to estimate gene expression in both transcriptomes. The 20 most expressed unigenes of eye and ventral skin transcriptomes are shown in the **S3 Table**. For the eye transcriptome, several actors involved in light perception where highlighted (*e*.*g*., rhodopsin, Gt protein and crystallins). Within the 20 most expressed unigenes of the ventral skin transcriptome, genes such as katanin (*i*.*e*., microtubule-severing protein), keratin and elongation factors are specifically represented. Several common genes, potentially expressed in hematocytes, were highlighted in both transcriptomes (*e*.*g*., ferritin and hemoglobin). Unsurprisingly, some mitochondrial genes (cytochrome oxidase, NADH dehydrogenase, cytochrome)—linked to eukaryotic energetic metabolism—are highly expressed in both transcriptomes.

### Opsin gene identification, sequence analyses, phylogeny and comparative gene expression

**Fig 6:**
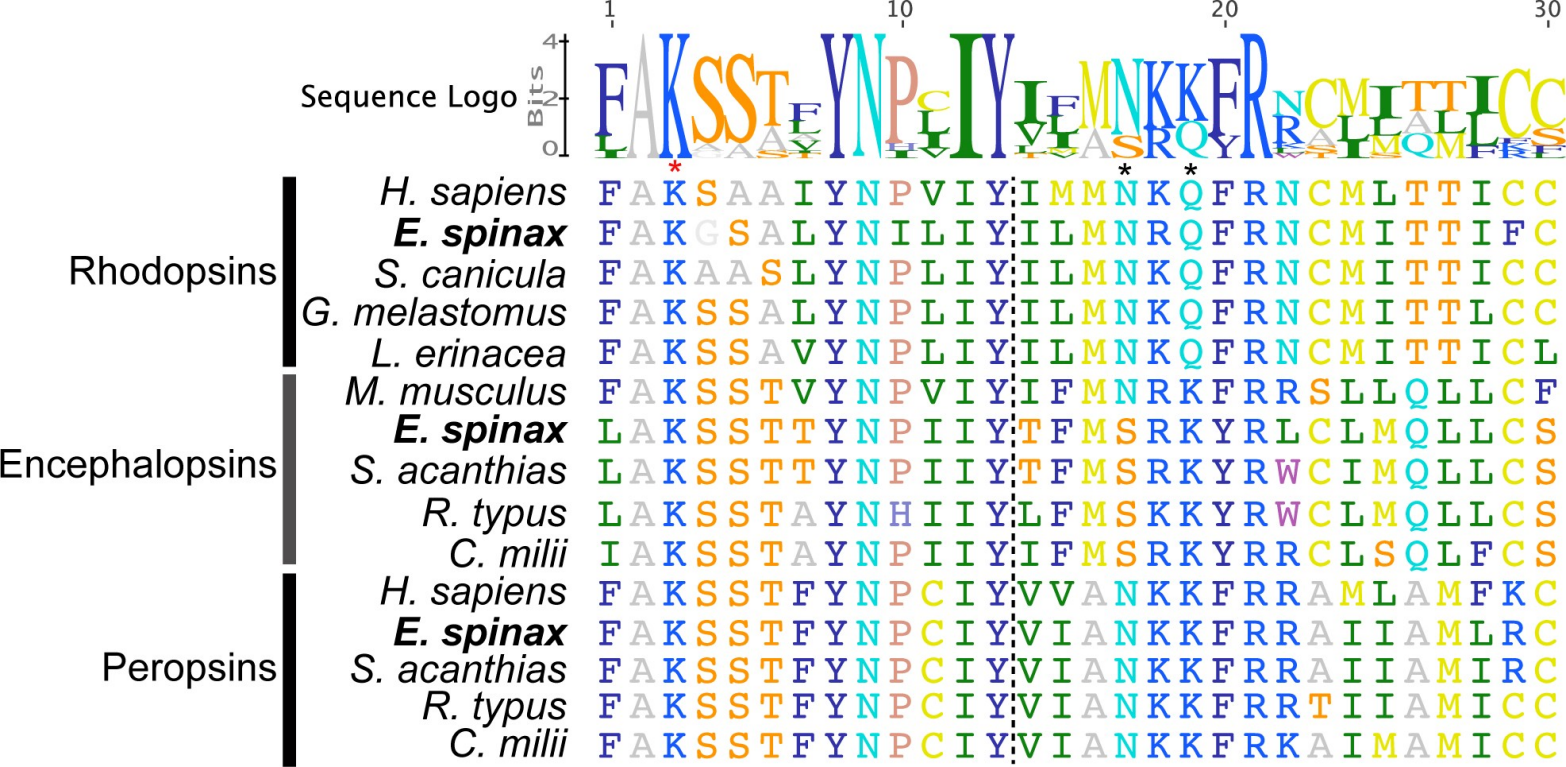
Amino acid alignment of members of three opsin types found in *E*. *spinax* transcriptomes. The selected alignment localizes to the border (vertical dotted line) between the 7^th^ transmembrane domain and the C-terminal tail. The alignment also includes reference opsins for other metazoans Red asterisk demarcates the position of the lysine residue critical for Schiff base formation (*i*.*e*., K296 of the *H*. *sapiens* rhodopsin). The black frame indicates the “NPxxY(x)_6_F” pattern containing the amino acid triad, highlighted with black asterisks (*i*.*e*., positions 310–312 in *H*. *sapiens* rhodopsin). The “NxQ” motif within the amino acid triad is classically observed in visual c-opsins but is not conserved in encephalopsins. *S*. *acanthias* (*Squalus acanthias* (encephalopsin: HAGU01045094.1, peropsin: HAGW01023913.1), *R*. *typus*: *Rhincodon typus* (encephalopsin: XP_020368171.1, peropsin: XP_020384809), *H*. *sapiens*: *Homo sapiens* (rhodopsin: NP000530.1, peropsin: NP006574.1), *L*. *erinacea*: *Leucoraja erinacea* (rhodopsin: P79863.1), *M*. *musculus*: *Mus musculus* (encephalopsin: AAD32670.1), *C*. *milli*: *Callorhinchus milli* (encephalopsin: XP_007892106.1, peropsin: XP_007895211), *G*. *melastomus*: *Galeus melastomus* (rhodopsin: O93441), *S*. *canicula*: *Scyliorhinus canicula* (rhodopsin: O93459.1), *C*. *conger*: *Conger conger*.

Sequences corresponding to three predicted opsins were found in the *E*. *spinax* pooled transcriptome. The sequences were translated into protein sequence with the ExPASy translate tool (ExPASy, Bioinformatics Resource Portal; http://web.expasy.org/translate). Reciprocal BLAST analyses revealed that the sequences matched to a rhodopsin, a peropsin and an encephalopsin (top blast results and the E-value of the hit concerning the reciprocal blast are listed in the **S1 Table**). These sequences were named accordingly: Es-rhodopsin (complete sequence), Es-peropsin (partial sequence) and Es-encephalopsin (complete sequence). The predicted proteins have molecular weights of 39,654.41 Da, 18,780.12 Da and 46,101.23 Da respectively. Using the MENSAT online tool, characteristic transmembrane domains were highlighted in all three sequences. We found very similar opsin sequences (*i*.*e*., encephalopsin and peropsin) in recent transcriptome data from *Squalus acanthias* [79]. In a comparative perspective, the *S*. *acanthias* sequences were added to the **Fig 6**. Comparison of the amino acid sequences of *E*. *spinax* and metazoan opsins demonstrated that the critical residues involved in the maintenance of the tertiary structure of the opsin molecule are present. These key sites include: (*i*) a conserved lysine residue (K) present in all three Es-opsins and localized at a position equivalent to K296 of the *H*. *sapiens* rhodopsin (position 284 for human peropsin, position 299 for human encephalopsin; see **S4–S6 Figs**) that is covalently linked to the chromophore via a Schiff base [80]; (*ii*) two conserved cysteine (C) residues involved in disulphide bond formation, localized at positions equivalent to C110 and C187 of human rhodopsin (C98 and C175 for human peropsin, C114 and C188 for human encephalopsin) and present in all Es-opsins [81] which are also conserved throughout the rest of the vertebrate opsin class; (*iii*) a conserved glutamate residue (E) at a position equivalent to 113 of the human rhodopsin that provides the negative counterion to the proton of the Schiff base [82] is also found in Es-rhodopsin; (*iv*) a conserved glutamate (E) at a position equivalent to E134 of the human rhodopsin (E138 of human encephalopsin) and providing a negative charge to stabilize the inactive opsin molecule [83] is present in Es-rhodopsin and Es-encephalopsin; (*vii*) the conserved glycosylation sites at positions equivalent to N2 and N15 of the human rhodopsin [84] are also present in Es-rhodopsin (see legends of the **Fig 6 and S4–S6 Figs** for more details). Although they are present in both Rh1 and Rh2 opsins of the elephant shark *C*. *milii*, the two conserved cysteine (C) residues at putative palmitoylation positions equivalent to C322 and C323 of the human rhodopsin [85] are not conserved in Es-rhodopsin. The trimmed alignment presented on the **Fig 6** focuses on the 7^th^ transmembrane domain and the C-terminal tail. It also highlights the “NPxxY(x)_6_F” pattern containing the amino acid triad (positions 310–312 in *H*. *sapiens* rhodopsin). The “NxQ” motif within the amino acid triad is classically observed in visual c-opsins but is not conserved in encephalopsins.

**Fig 7:**
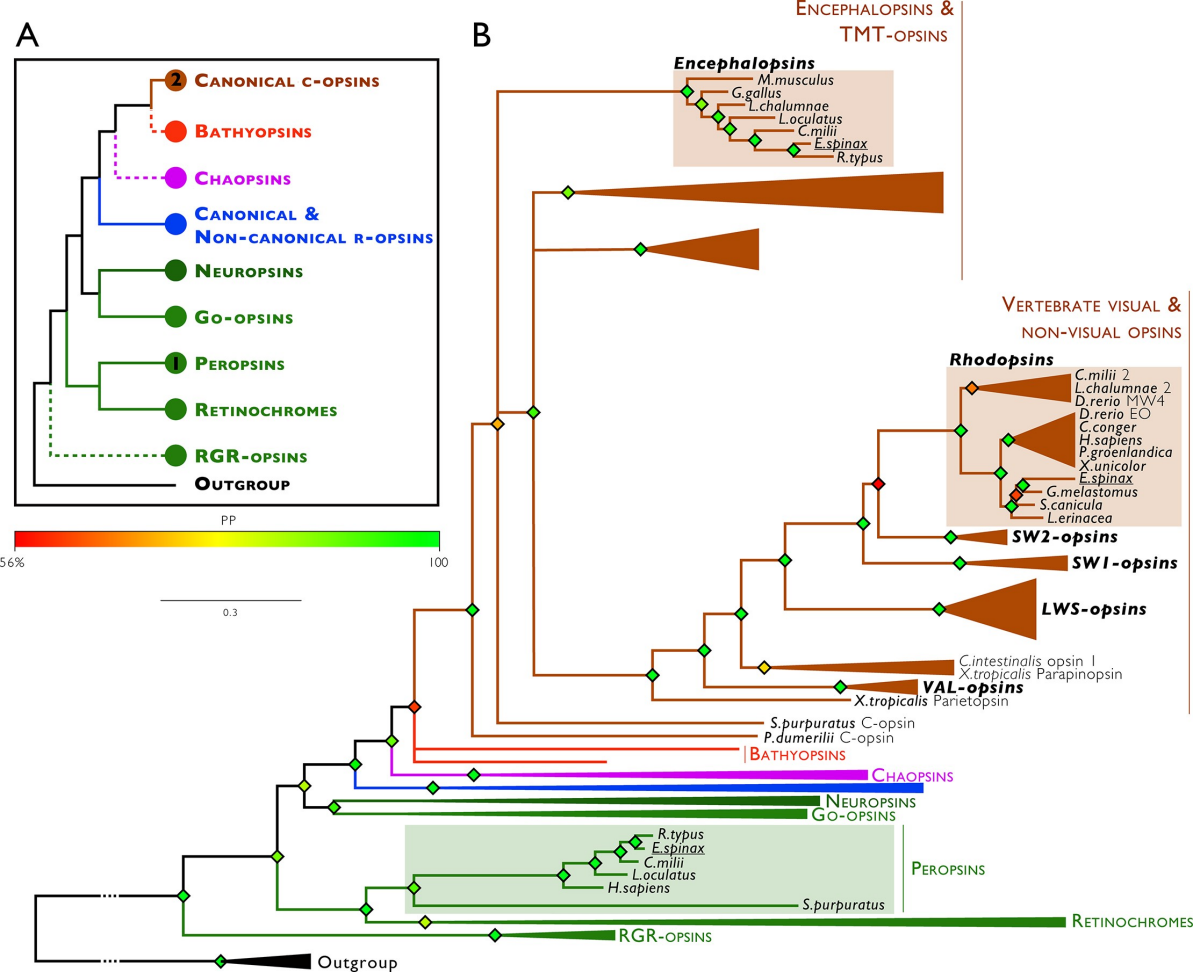
Metazoan opsin phylogenetic tree including the *E*. *spinax* opsins. Predicted *E*. *spinax* opsin proteins were included in a large opsin phylogeny (*i*) to ensure their opsin status and (*ii*) define their belonging to known classical opsin groups. Phylogeny was constructed using the Bayesian method (MrBayes software, v.3.2.2). Branch support values are indicated by color-codes next to the branching points and correspond to posterior probabilities. Branch length scale bar indicates relative amount of amino acid change. C-opsins: Ciliary opsins, R-opsins: Rhabdomeric opsins, RGR opsin: Retinal G-protein coupled receptors, Outgroup (black): melatonin receptor.

The sequences of the predicted opsins of *E*. *spinax* were then incorporated in a phylogenetic analysis of metazoan opsins. The constructed tree validated the classification of *E*. *spinax* predicted opsins into the ciliary opsin group for the Es-rhodopsin (vertebrate visual opsins) and the Es-encephalopsin (vertebrate extraocular opsin, opsin 3 group). Es-Peropsin was also confirmed to belong to peropsin/RGR-opsin group with a clear clustering with vertebrate peropsins. Confidence in this classification is high due to the high posterior probabilities values (**Fig 7**).

### Phototransduction and “light interacting toolkit” genes identification

**Fig 8:**
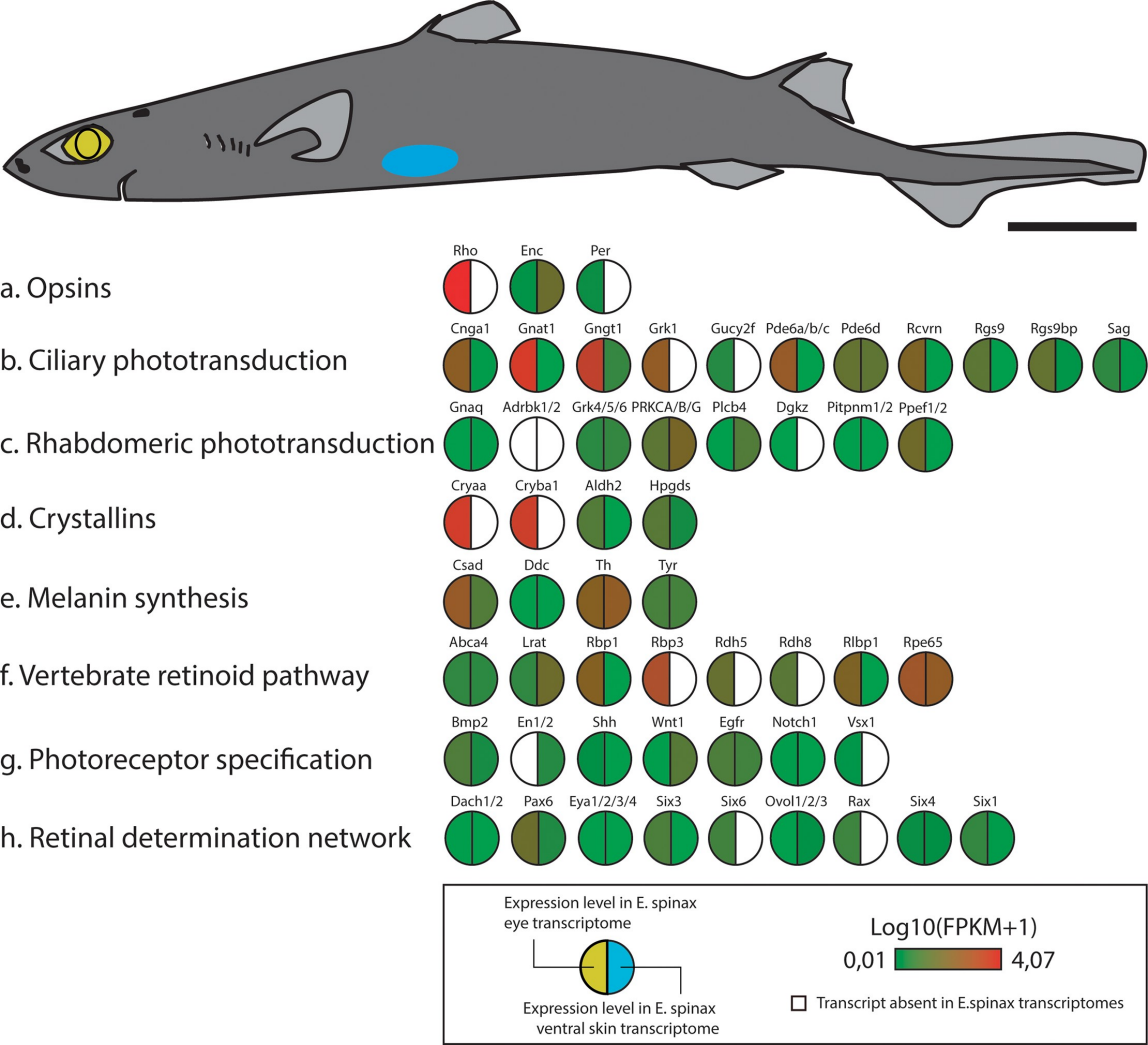
Predicted light-interacting toolkit genes within *E*. *spinax* eye and ventral skin.

An analysis of the *E*. *spinax* transcriptome generated from the eye and ventral skin tissues of *E*. *spinax* revealed transcripts encoding proteins with high similarities to the key components of visual transduction cascades. We identified genes encoding putative opsin photopigments and proteins involved in subsequent activation and deactivation of the cascades **(Fig 8)**.

In addition to the expression of the Es-rhodopsin, ciliary phototransduction actors such as the GTP-binding transducin (*i*.*e*., Gnat1, Gngt1), the phosphodiesterase 6 (*i*.*e*., Pde6a/b/c/d), the cGMP-gated cation channels (*i*.*e*., Cnga1), the retinal guanylyl cyclase 2 (*i*.*e*., Gucy2f), the rhodopsin kinase (*i*.*e*., Grk1), the arrestin (*i*.*e*., Sag), the recoverin (*i*.*e*., Rcvrn) and the regulator of G protein signaling 9 (*i*.*e*., Rgs9) are preferentially expressed in *E*. *spinax* eye transcriptome. Guanine nucleotide-binding protein Gi/Go/Gs expression is not restricted to the eye transcriptome while a clear eye-specific expression is observed for the transducin (*i*.*e*., Gnat1, Gngt1).

No rhabdomeric opsin was highlighted in the *E*. *spinax* reference transcriptome and no clear expression trend is observed for potential actors of the rhabdomeric phototransduction.

Crystallins are specifically expressed in *E*. *spinax* eye transcriptome (*i*.*e*., several isoforms of Alpha-crystallins Cryaa and Beta-crystallins Cryba1). Genes associated to other light related processes and obtained from the Light-Interaction Toolkit (LIT 1.0) [38], such as melanin synthesis actors, vertebrate retinoid pathway actors, photoreceptor specification actors, retinal determination network actors and diurnal clock actors were also found to be expressed in both tissues **(Fig 8 and S1 Table)**.

Several actors of the vertebrate retinoid pathway are specifically expressed at the level of the eye such as the retinol-binding protein 1 and 2 (*i*.*e*., Rbp1, 3), the retinol dehydrogenase 5 and 8 (*i*.*e*., Rdh5, Rdh8) and the retinaldehyde binding protein 1 (*i*.*e*., Rlbp1)

### Encephalopsin immunodetections

**Fig 9:**
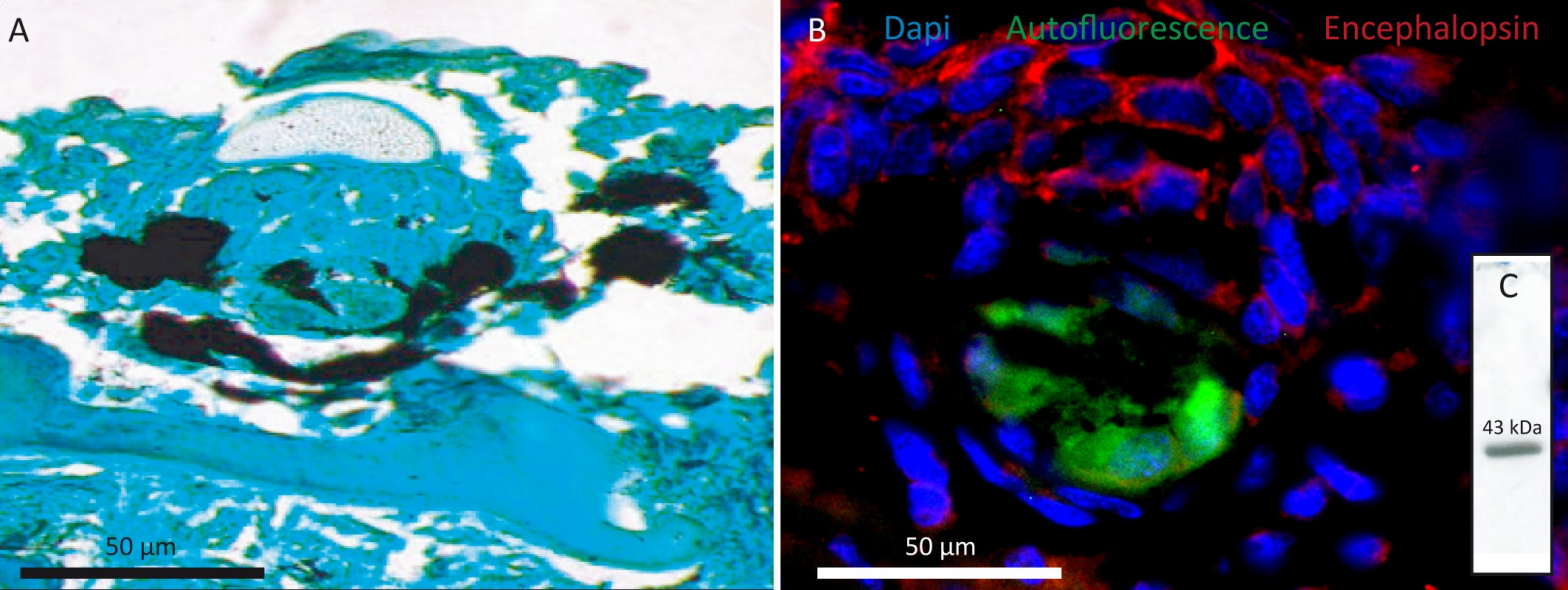
Encephalopsin immunodetection in *E*. *spinax*. A. photosensitive film of immunoblotting performed on the protein extract of *E*. *spinax* ventral and dorsal skin as well as retina with an antibodies directed against extraocular opsin: anti-encephalopsin PAb from Genetex, GTX 70609, lot number 821 400 929, 1/2000. 50 **μ**g of total protein were used in each well. B. Cryosection immunofluorescence directed against extraocular opsins in different tissues of the lanternshark, *E*. *spinax*. Visualization of a photophore paraffin section (A). Visualization of the labelling on cryosections of a ventral skin section with photophores (B), a section of the retina (C). The B and C sections were given the primary antibody GTX (primary antibody: anti-encephalopsin PAb from Genetex, GTX 70609, lot number 821 400 929, 1/50). The secondary antibody was coupled with a red fluorochrome (Alexa Fluor 594 Goat Anti-Rabbit IgG (H+L) Antibody, highly cross-adsorbed (A-11037), 1/300 from Life Technologies Limited). C, conjunctive tissue; E, epidermis; Ir: iris-like structure related pigmented cell; L, lens cell; Ph: photocyte; Ps: pigmented sheath; D: dermal denticle; R: rod, C: cone layer. Scale bar: 50 **μ**m.

The encephalopsin protein sequence predicted in this study based on RNA-seq data appears highly similar to other vertebrate orthologous encephalopsins. It shares 52% of identity and 61% of similarity with human encephalopsin (see **S6 Fig**). Based on this similarity, a commercial anti-encephalopsin (*H*. *sapiens*) antibody was selected for immunodetections. On *E*. *spinax* ventral skin sections, a strong anti-encephalopsin immunoreactivity was observed at the level of the cell membrane of the epidermal cells and of pigmented cells related to the iris-like structure (**Fig 9A and 9B**). Similarly, the cells on the surface of the lens were labelled. Photocyte autofluorescence is visible in **Fig 9B** (in green). The dorsal skin showed a weaker immunoreactivity of the cell membranes of the epidermal cells while no staining was observed in the retina (data not shown). Control with omission of the primary antibody did not show any non-specific binding of the secondary antibodies (data not shown).

Immunoblot analyses revealed a strong immunoreactive band in the extract of shark ventral skin tissues labelled using the anti-encephalopsin antibody (**Fig 9C**). This band corresponds to a protein with an apparent molecular weight of 43kDa matching the mass of the predicted encephalopsin protein (*e*.*g*., opsins generally have a molecular weight comprised between 39 and 45 kDa [86]). The protein extract from the dorsal skin showed a similar immunoreactivity pattern (data not shown). Finally, no labelling could be detected in the retina of this shark (data not shown).

## Discussion

This study presents the first release of a protein-coding transcriptome for the lanternshark *E*. *spinax*. The transcriptome sequences of *E*. *spinax* were assembled *de novo* and tissue-specific abundance of transcripts has been visualized. This study does not represent a proper differential expression data as no transcriptome replication has been performed. However, gene expression trends can be observed between ventral skin and eye transcriptomes. These large-scale NGS data have a high percentage of significant hits with the NR public database. The high completeness of these single tissue transcriptomes was confirmed by the presence of 98% of orthologous genes of the « Core eukaryotic gene dataset ».

Efforts were made toward the identification of genes putatively involved in light perception, mainly. The Es-rhodopsin and Es-peropsin mRNA were found exclusively in the eye transcriptome. Based on these observations and on the literature, it seems clear that the rhodopsin and peropsin are functionally coupled as previously described, which also confirm the monochromatic vision of the species.

Conversely, the Es-encephalopsin was found in both tissues but with a much higher expression in ventral skin (based on FPKM values) **(Fig 8).** Vertebrate encephalopsins belong to the OPN3 that are non-visual opsins that have been identified in the brain of vertebrate and invertebrates. OPN3 also contain TMT (teleost multiple tissue) opsins in teleosts, pteropsins in insects and c-opsins in annelids [87–89]. In vertebrates, encephalopsin is expressed in a variety of extra-retinal tissues such as brain, testes or skin as well as within the retina. Haltaufderhyde *et al*. [90] suggested that encephalopsin might initiate light–induced signaling pathways contributing to UVR phototransduction in skin. Sety *et al*. [91] showed that skin encephalopsin senses blue light in the solar spectrum and activate a pathway leading to radiation-induced skin hyperpigmentation.

## Conclusion

Compared with laborious “gene by gene” analyzes (*e*.*g*., [92]), next-generation sequencing (NGS) technologies allow obtaining a deeper and more complete view of transcriptomes [93]. For non-model or emerging model marine organisms, NGS technologies offer a great opportunity for rapid access to genetic information. Our study presents the first transcriptomes of the lanternshark *E*. *spinax* opening a window on a better understanding of the biology of this species.

In the context of the opsin-based perception of light, the characterization of the *E*. *spinax* eye transcriptome revealed the presence of the unique visual opsin (Es-rhodopsin) most probably functionally coupled with a peropsin (Es-peropsin). Investigation of ventral skin transcriptome of the lanternshark *E*. *spinax* revealed the extraocular expression of an encephalopsin, *i*.*e*. a non-visual ciliary opsin (Es-encephalopsin). Immunodetections of the encephalopsin showed a widespread expression within the cell membrane of the shark epidermal cells surrounding the photophore while no expression was seen in the photocytes themselves. Where darkness is permanent, bioluminescence constitutes the main source of light and these sharks are no exception to the rule. These mid-water cartilaginous fishes indeed emit a ventral light to efficiently mask their silhouette from downwelling ambient light and remain hidden from predators and preys [94]. The encephalopsin expression in the surrounding area of the photophore supports the hypothesis of a potential interaction between light emission and reception. This hypothesis should be confirmed by a deeper characterisation of the *E*. *spinax* encephalopsin expression and function.

All together, the data generated within this study represent an important contribution to the existing genomic resources for shark taxa and should help research projects on lanternsharks by providing a valuable tool.

## Supporting information

S1 Table Search for opsins and “light interacting genes” in the *E*. *spinax* eye and ventral skin transcriptomes based on reference sequences. Homologues to ciliary and rhabdomeric phototransduction components, crystallins, melanin synthesis components, vertebrate retinoid pathway components, photoreceptor specification actors, retinal determination network actors, invertebrate retinoid pathway and diurnal clock components and their reciprocal best BLAST hit in *E*. *spinax* transcriptomes. BLAST analyses were also performed on *Rhyncodon typus* [93] and *Callorhinchus milii* [45, 46, 97] genomes. FPKM values and fold change (log10) used for the Fig 8 are shown.
(XLSX)

S2 Table Reference genes used for the phylogenetic analysis. Opsins and melatonin receptors opsins from various organisms used as references for the phylogenetic Bayesian analysis.
(XLSX)

S3 Table Blastn/x of 10 most expressed Unigenes in ventral skin and eye transcriptomes of *E*. *spinax*. Unigene commonly found in both transcriptome hits are in bold.
(XLSX)

S1 Fig Distributions of contigs and unigenes sizes in *E*. *spinax* retina and ventral skin transcriptomes. The length of contigs and unigenes ranged from 200 bp to more than 3,000 bp. Each range is defined as follows: sequences within the range of X are longer than X bp but shorter than Y bp.
(PDF)

S2 Fig (A) E-value distributions, (B) similarity distributions and (C) species distributions of the top BLAST hits for all unigenes from *E*. *spinax* transcriptomes in the NR database.
(PDF)

S3 Fig Gene ontology classifications of assembled unigenes *E*. *spinax*. The results are summarized in three main categories: Biological process, cellular component and molecular function.
(PDF)

S4 Fig Amino acid alignment of *E*. *spinax* rhodopsin with reference metazoan rhodopsins. (PDF)

S5 Fig Amino acid alignment of *E*. *spinax* peropsin with reference metazoan peropsins. (PDF)

S6 Fig Amino acid alignment of *E*. *spinax* encephalopsin with reference metazoan encephalopsins. (PDF)
