# Supplementary material for: De novo transcriptome analyses provide insights into opsin-based photoreception in the lanternshark Etmopterus spinax
Source: PLoS One. 2018 Dec 31;13(12):e0209767. doi: 10.1371/journal.pone.0209767 (PMC6312339; doi:10.1371/journal.pone.0209767)
Supplement: S1 Fig — The length of contigs and unigenes ranged from 200 bp to more than 3,000 bp. Each range is defined as follows: sequences within the range of X are longer than X bp but shorter than Y bp. (PDF) [file pone.0209767.s004.pdf]

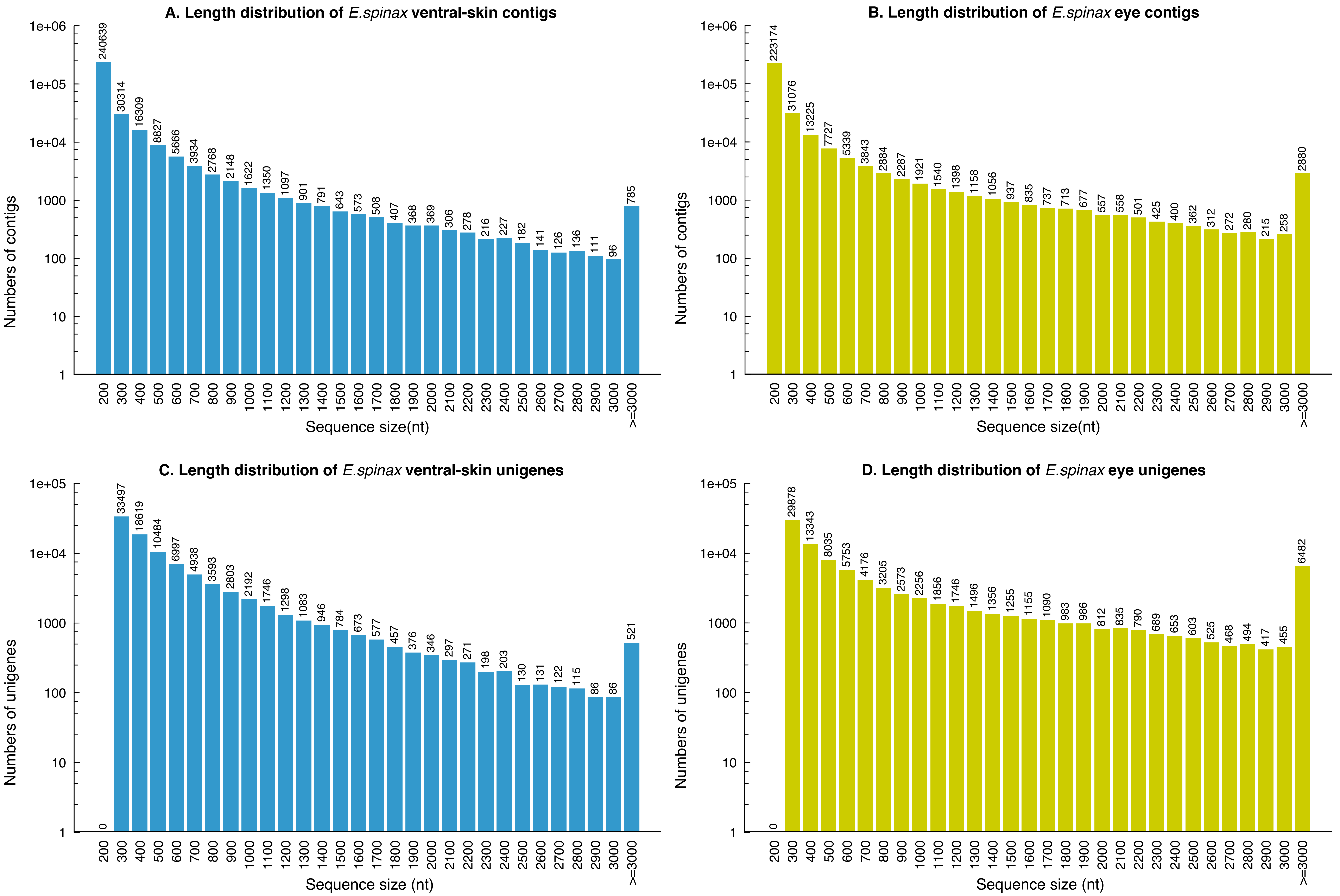
A. Length distribution of E.spinax ventral-skin contigs
Numbers of contigs
1e+06
1e+05
1e+04
1000
100
10
1
240639
30314
16309
8827
5666
3934
2768
2148
1622
1350
1097
901
791
643
573
508
407
368
369
306
278
216
227
182
141
126
136
111
96
785
200 300 400 500 600 700 800 900 1000 1100 1200 1300 1400 1500 1600 1700 1800 1900 2000 2100 2200 2300 2400 2500 2600 2700 2800 2900 3000 >=3000
Sequence size(nt)
B. Length distribution of E.spinax eye contigs
Numbers of contigs
1e+06
1e+05
1e+04
1000
100
10
1
223174
31076
13225
7727
5339
3843
2884
2287
1921
1540
1398
1158
1056
937
835
737
713
677
557
558
501
425
400
362
312
272
280
215
258
2880
200 300 400 500 600 700 800 900 1000 1100 1200 1300 1400 1500 1600 1700 1800 1900 2000 2100 2200 2300 2400 2500 2600 2700 2800 2900 3000 >=3000
Sequence size(nt)
C. Length distribution of E.spinax ventral-skin unigenes
Numbers of unigenes
1e+05
1e+04
1000
100
10
1
0
33497
18619
10484
6997
4938
3593
2803
2192
1746
1298
1083
946
784
673
577
457
376
346
297
271
198
203
130
131
122
115
86
86
521
200 300 400 500 600 700 800 900 1000 1100 1200 1300 1400 1500 1600 1700 1800 1900 2000 2100 2200 2300 2400 2500 2600 2700 2800 2900 3000 >=3000
Sequence size (nt)
D. Length distribution of E.spinax eye unigenes
Numbers of unigenes
1e+05
1e+04
1000
100
10
1
0
29878
13343
8035
5753
4176
3205
2573
2256
1856
1746
1496
1356
1255
1155
1090
983
986
812
835
790
689
653
603
525
468
494
417
455
6482
200 300 400 500 600 700 800 900 1000 1100 1200 1300 1400 1500 1600 1700 1800 1900 2000 2100 2200 2300 2400 2500 2600 2700 2800 2900 3000 >=3000
Sequence size (nt)
