# Supplementary figures and images for: De novo transcriptome analyses provide insights into opsin-based photoreception in the lanternshark Etmopterus spinax
Source: PLoS One. 2018 Dec 31;13(12):e0209767. doi: 10.1371/journal.pone.0209767 (PMC6312339; doi:10.1371/journal.pone.0209767)

Taxonomic distribution

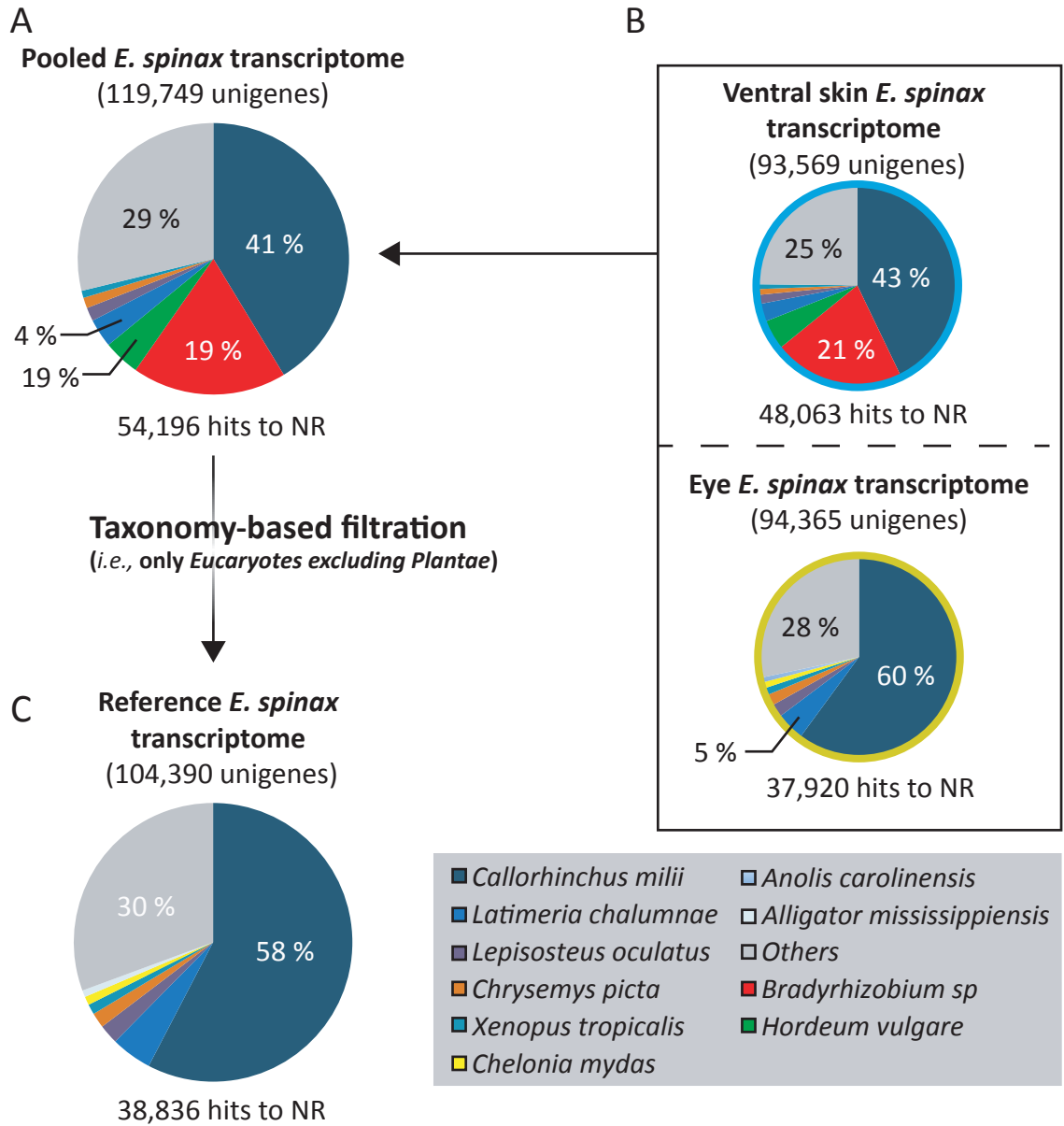

E-value distribution

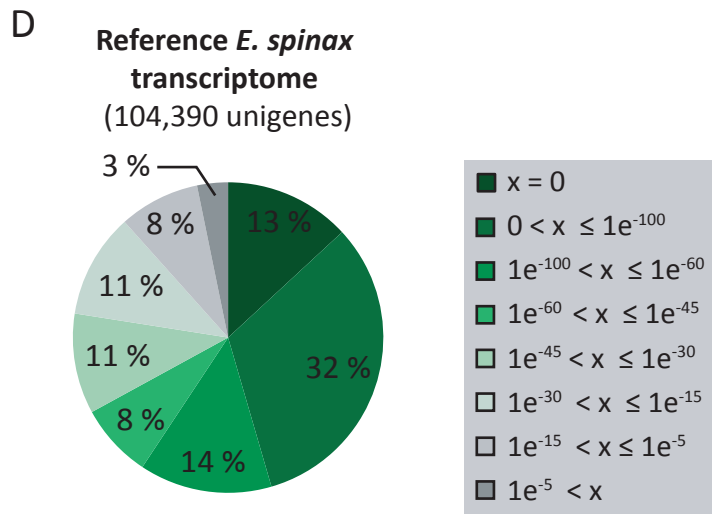

Supplement: S2 Fig — (A) E-value distributions, (B) similarity distributions and (C) species distributions of the top BLAST hits for all unigenes from E. spinax transcriptomes in the NR database. (PDF) [file pone.0209767.s005.pdf]

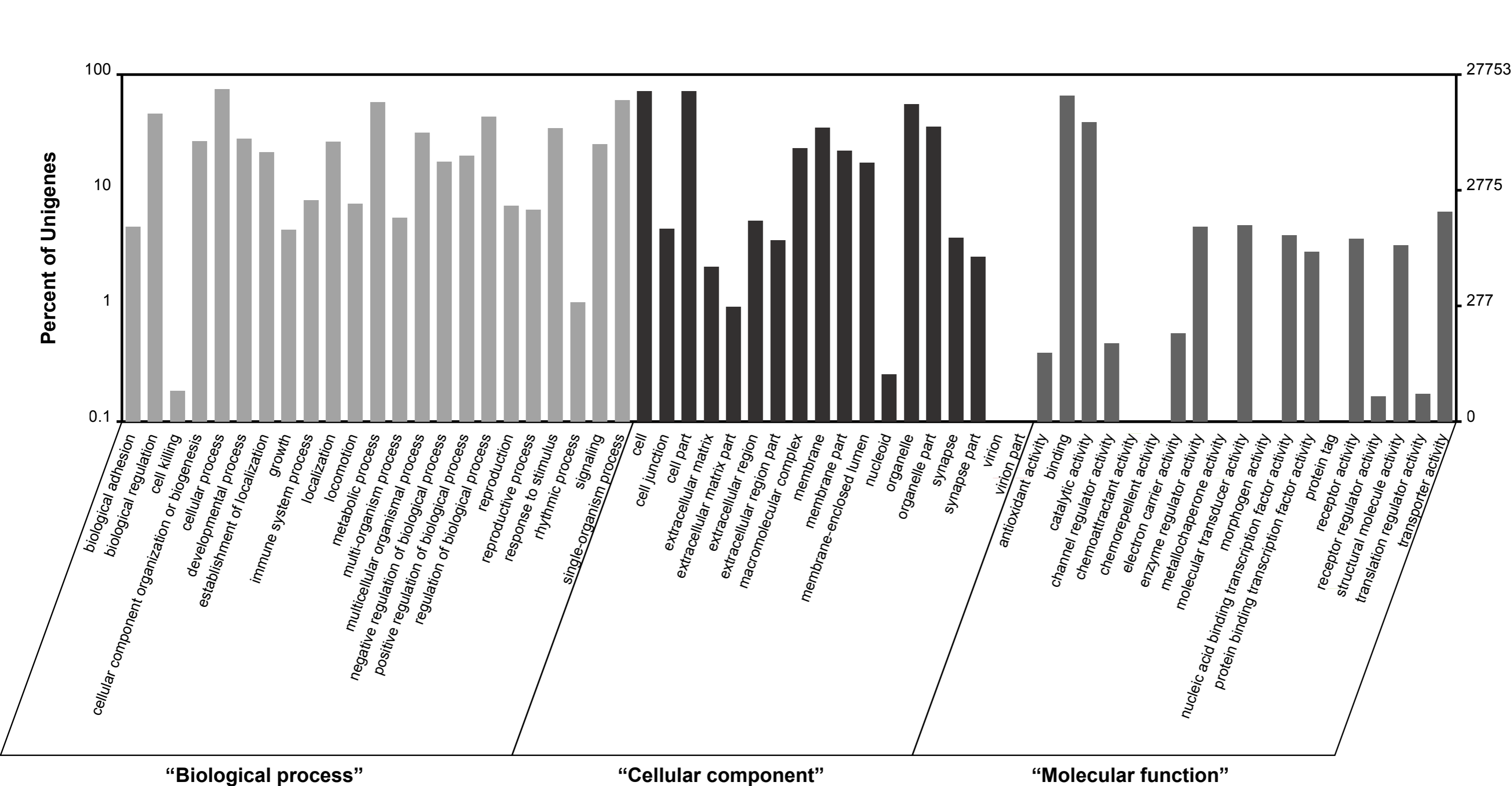

Supplement: S3 Fig — The results are summarized in three main categories: Biological process, cellular component and molecular function. (PDF) [file pone.0209767.s006.pdf]

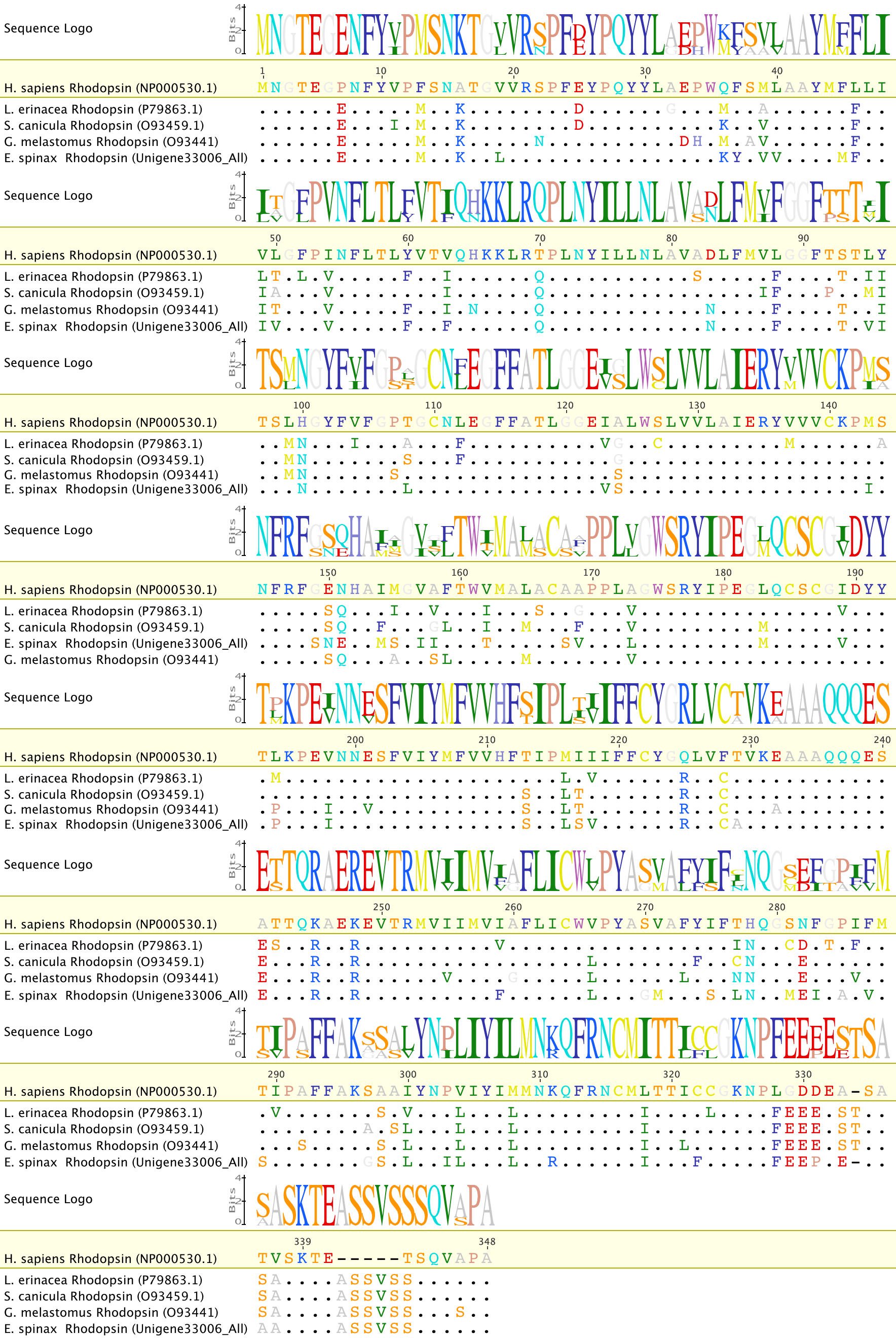

Supplement: S4 Fig — (PDF) [file pone.0209767.s007.pdf]

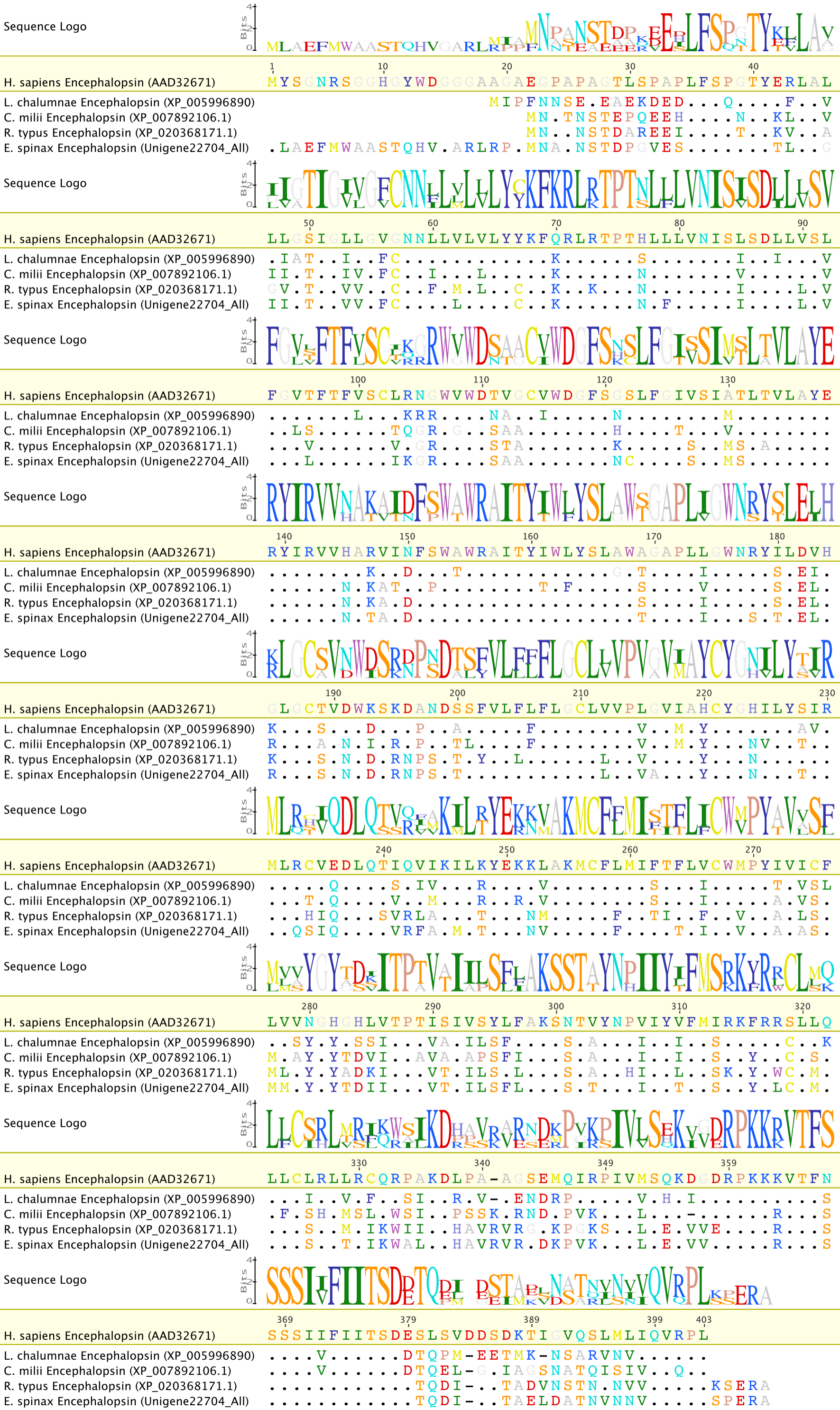

Supplement: S6 Fig — (PDF) [file pone.0209767.s009.pdf]
