# Supplementary material for: De novo transcriptome analyses provide insights into opsin-based photoreception in the lanternshark Etmopterus spinax
Source: PLoS One. 2018 Dec 31;13(12):e0209767. doi: 10.1371/journal.pone.0209767 (PMC6312339; doi:10.1371/journal.pone.0209767)
Supplement: S5 Fig — (PDF) [file pone.0209767.s008.pdf]

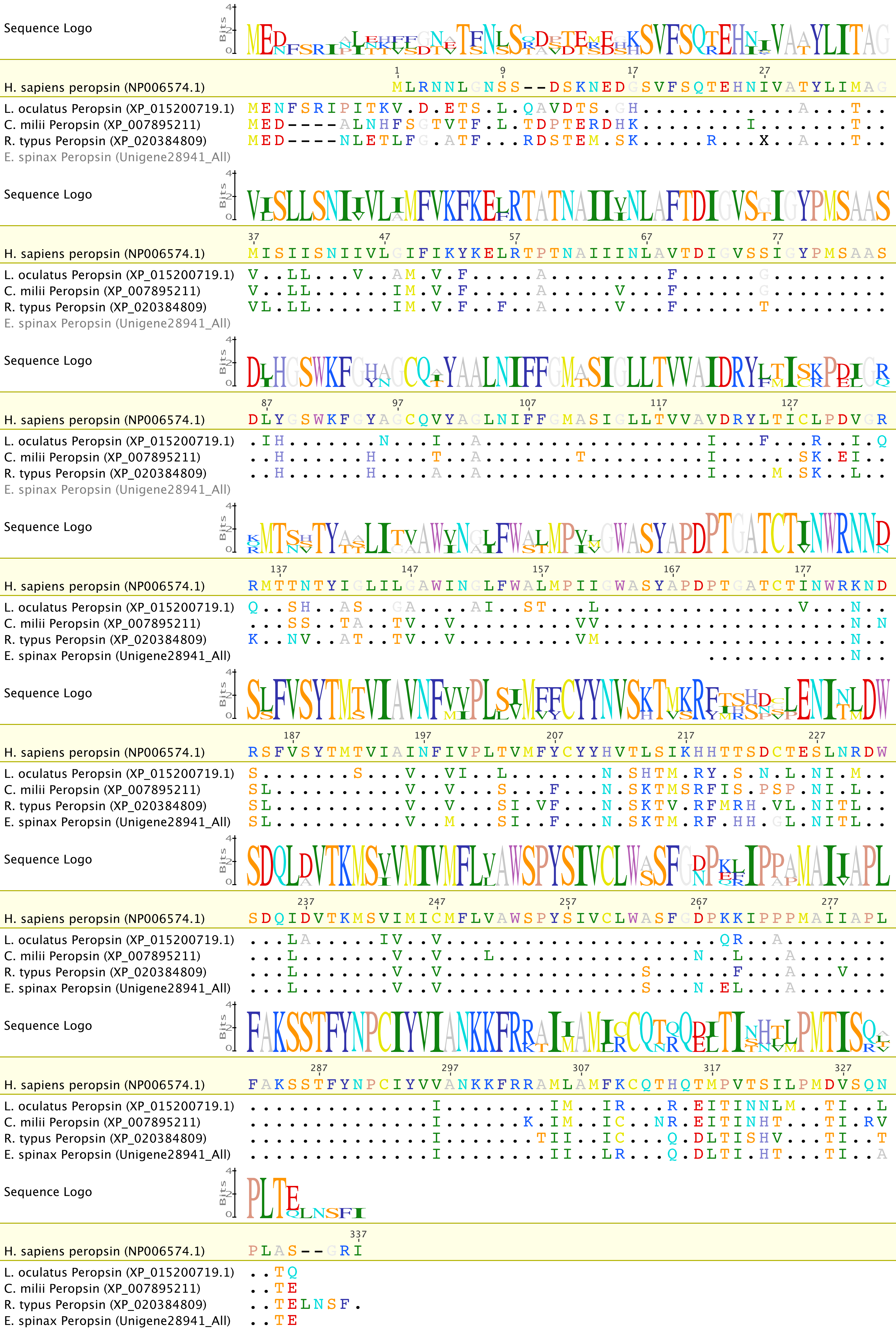
Sequence Logo
H. sapiens peropsin (NP006574.1)
1 9 17 27
MLRNNLGNSS--DSKNEDGSVFSQTEHNIVATYLIMAG
L. oculatus Peropsin (XP_015200719.1)
MENFSRIPITKV.D.ETS.L.QAVDTS.GH.............A...T..
C. milii Peropsin (XP_007895211)
MED----ALNHFSGTVTF.L.TDPTERDHK........I........T..
R. typus Peropsin (XP_020384809)
MED----NLETLFG.ATF...RDSTEM.SK.....R...X..A...T..
E. spinax Peropsin (Unigene28941_All)
Sequence Logo
H. sapiens peropsin (NP006574.1)
37 47 57 67 77
MISIISNIIVLGIFIKYKELRTPTNAIIINLAVTDIGVSSIGYPMSAAS
L. oculatus Peropsin (XP_015200719.1)
V..LL...V..AM.V.F.....A.........F......G..........
C. milii Peropsin (XP_007895211)
V..LL......IM.V.F.....A......V...F......G..........
R. typus Peropsin (XP_020384809)
VL.LL......IM.V.F..F..A......V...F......T..........
E. spinax Peropsin (Unigene28941_All)
Sequence Logo
H. sapiens peropsin (NP006574.1)
87 97 107 117 127
DLYGSWKFGYAGCQVYAGLNIFFGMASIGLLTVVAVDRYLTICLPDVGR
L. oculatus Peropsin (XP_015200719.1)
.IH.......N...I..A...................I...F...R..I.Q
C. milii Peropsin (XP_007895211)
..H.......H....T..A........T.........I......SK.EI..
R. typus Peropsin (XP_020384809)
..H.......H....A..A..................I.....M.SK..L..
E. spinax Peropsin (Unigene28941_All)
Sequence Logo
H. sapiens peropsin (NP006574.1)
137 147 157 167 177
RMTTNTYIGLILGAWINGLFWALMPIIGWASYAPDPTGATCTINWRKND
L. oculatus Peropsin (XP_015200719.1)
Q..SH..AS..GA....AI..ST...L................V...N..
C. milii Peropsin (XP_007895211)
...SS..TA..TV..V..........VV..................N.N
R. typus Peropsin (XP_020384809)
K..NV..AT..TV..V..........VM..................N..
E. spinax Peropsin (Unigene28941_All)
.............N..
Sequence Logo
H. sapiens peropsin (NP006574.1)
187 197 207 217 227
RSFVSYTMTVIAINFIVPLTVMFYCYYHVTLSIKHHTTSDCTESLNRDW
L. oculatus Peropsin (XP_015200719.1)
S.......S...V..VI..L........N.SHTM.RY.S.N.L.NI.M..
C. milii Peropsin (XP_007895211)
SL..........V..V...S...F...N.SKTMSRFIS.PSP.NI.L..
R. typus Peropsin (XP_020384809)
SL..........V..V...SI.VF...N.SKTV.RFMRH.VL.NITL..
E. spinax Peropsin (Unigene28941_All)
SL..........V..M...SI..F...N.SKTM.RF.HH.GL.NITL..
Sequence Logo
H. sapiens peropsin (NP006574.1)
237 247 257 267 277
SDQIDVTKMSVIMICMFLVAWSPYSIVCLWASFGDPKKIPPPMAIIAPL
L. oculatus Peropsin (XP_015200719.1)
...LA.....IV..V.....................QR..A........
C. milii Peropsin (XP_007895211)
...L......V..V...L..................N..L...A......
R. typus Peropsin (XP_020384809)
...L......V..V.............S......F...A...V...
E. spinax Peropsin (Unigene28941_All)
...L......V..V.............S...N.EL..A......
Sequence Logo
H. sapiens peropsin (NP006574.1)
287 297 307 317 327
FAKSSTFYNPCIYVVANKKFRRAMLAMFKCQTHQTMPVTSILPMDVSQN
L. oculatus Peropsin (XP_015200719.1)
...............I.......IM..IR...R.EITINNLM..TI..L
C. milii Peropsin (XP_007895211)
...............I......K.IM..IC..NR.EITINHT..TI.RV
R. typus Peropsin (XP_020384809)
...............I.....TII..IC...Q.DLTISHV..TI..T
E. spinax Peropsin (Unigene28941_All)
...............I......II..LR...Q.DLTI.HT..TI..A
Sequence Logo
H. sapiens peropsin (NP006574.1)
337
PLAS--GRI
L. oculatus Peropsin (XP_015200719.1)
..TQ
C. milii Peropsin (XP_007895211)
..TE
R. typus Peropsin (XP_020384809)
..TELNSF.
E. spinax Peropsin (Unigene28941_All)
..TE
